# Ancient DNA from a lost Negev Highlands desert grape reveals a Late Antiquity wine lineage

**DOI:** 10.1073/pnas.2213563120

**Published:** 2023-04-17

**Authors:** Pnina Cohen, Roberto Bacilieri, Jazmín Ramos-Madrigal, Eyal Privman, Elisabetta Boaretto, Audrey Weber, Daniel Fuks, Ehud Weiss, Tali Erickson-Gini, Scott Bucking, Yotam Tepper, Deborah Cvikel, Joshua Schmidt, M. Thomas P. Gilbert, Nathan Wales, Guy Bar-Oz, Meirav Meiri

**Affiliations:** ^a^The Steinhardt Museum of Natural History and Israel National Center for Biodiversity Studies, Tel Aviv University, Tel Aviv 6997801, Israel; ^b^UMR Amélioration génétique et adaptation de plantes Institut, Univ Montpellier, Centre de Coopération Internationale en Recherche Agronomique pour le Développement, Institut national de recherche pour l’agriculture, l’alimentation et l’environnement, Institut Agro, F-34398 Montpellier, France; ^c^Faculty of Health and Medical Sciences, Center for Evolutionary Hologenomics, The Globe Institute, University of Copenhagen, 1353 Copenhagen, Denmark; ^d^Department of Evolutionary and Environmental Biology, Institute of Evolution, University of Haifa, Haifa 3498838, Israel; ^e^Max Planck-Weizmann Center for Integrative Archaeology and Anthropology, Dangoor Research Accelerator Mass Spectrometry Laboratory, Weizmann Institute of Science, Rehovot 7610001, Israel; ^f^Department of Archaeology, McDonald Institute for Archaeological Research, Cambridge CB2 3ER, UK; ^g^The Martin (Szusz) Department of Land of Israel Studies and Archaeology, Bar-Ilan University, 52900 Ramat Gan, Israel; ^h^Archaeological Division, Israel Antiquities Authority, 61012 Tel Aviv, Israel; ^i^Department of History, DePaul University, Chicago, IL 60614; ^j^Department of Archaeology, Zinman Institute of Archaeology, University of Haifa, Haifa 3498837, Israel; ^k^Department of Maritime Civilizations, University of Haifa, Mount Carmel, Haifa 3498838, Israel; ^l^The Leon Recanati Institute for Maritime Studies, University of Haifa, Mount Carmel, Haifa 3498838, Israel; ^m^Norwegian University of Science and Technology, University Museum, 7012 Trondheim, Norway; ^n^Department of Archaeology, University of York, York YO1 7EP, United Kingdom; ^o^School of Archaeology and Maritime Cultures, University of Haifa, Mount Carmel, Haifa 3498837, Israel

**Keywords:** archaeobotany, viticulture, Late Antiquity, Negev Highlands, ancient DNA

## Abstract

Recent excavations of Late Antiquity settlements in the Negev Highlands of southern Israel uncovered a society that established commercial-scale viticulture in an arid environment [D. Fuks *et al.*, *Proc. Natl. Acad. Sci. U.S.A.* **117**, 19780–19791 (2020)]. We applied target-enriched genome-wide sequencing and radiocarbon dating to examine grapevine pips that were excavated at three of these sites. Our analyses revealed centuries long and continuous grape cultivation in the Southern Levant. The genetically diverse pips also provided clues to ancient cultivation strategies aimed at improving agricultural productivity and ensuring food security. Applying genomic prediction analysis, a pip dated to the eighth century CE was determined to likely be from a white grape, to date the oldest to be identified. In a kinship analysis, another pip was found to be descendant from a modern Greek cultivar and was thus linked with several popular historic wines that were once traded across the Byzantine Empire. These findings shed light on historical Byzantine trading networks and on the genetic contribution of Levantine varieties to the classic Aegean landscape.

## Significance

The modern winemaking industry is heavily reliant on a limited number of European grape cultivars, which are best suited for cultivation in temperate climates. Global warming emphasizes the need for diversity in this high-impact agricultural crop. Grapevine lineages bred in hot and arid regions, often preserved over centuries, may present an alternative to the classic winemaking grape cultivars. Our study of a legacy grapevine variety from the Negev Highlands desert of southern Israel sheds light on its genetics, biological properties, and lasting impact. The modern-day close relatives of the archaeological grapes may now provide an exceptional platform for future studies on grapevine resilience to aridity.

The grapevine (*Vitis vinifera,* subsp. *vinifera*) plays a vital economic and cultural role worldwide. Since the domestication of the wild vine (*V. vinifera*, subsp. *sylvestris*) in Southwest Asia over 6,000 y ago (1, 2), it is been primarily grown for wine (3). Viticulture (grape growing) and viniculture (winemaking) evolved along multiple historical pathways in diverse wine regions and produced a myriad of legacy cultivars growing in their particular terroir.

Of the thousands of extant winemaking grape varieties, only 11 cultivars of European origin (Cabernet Sauvignon, Chasselas, Chardonnay, Grenache, Merlot, Monastrell, Pinot Noir, Riesling, Sauvignon Blanc, Syrah, and Ugni Blanc) cover more than a third of the winemaking vineyards worldwide (4–6). This remarkably small number of cultivars is grown across a relatively narrow geographic zone (4, 5) with generally fixed sets of climatic conditions, exposing the winemaking industry to stressors like global warming. A recent study found that a rise of 2 °C in worldwide median temperatures would devastate grapevine cultivation in more than half of the current winegrowing areas (6). This is supported by increasing evidence that temperature changes affect grapevine maturation time, impacting berry and wine quality (7). An increase in diversity would most likely mitigate this effect and may even reverse it (6). Thus, endemic grapevine varieties in arid regions offer a prospect of crop resilience to the changing climate.

The Negev Highlands region is a high plateau in southern Israel. It is characterized by aridity (aridity index ≤0.10; mean annual rainfall of 80 to 100 mm/y) (8), sever summer droughts, and variant diurnal (hot/cold) temperature. Historically, regional agriculturalists took advantage of the local geography and the predictability of seasonal rainfalls to harvest ample runoff rainwater for their crops. Recent excavations of urban Byzantine/Early Islamic settlements in the Negev Highlands exposed a desert society that existed in the fourth to ninth centuries CE and was sustained by sophisticated dryland agriculture that included a prosperous viticulture, particularly in the fifth to mid-sixth centuries (9). The wine produced in the Negev was traded overseas and achieved a formidable international reputation (10). Recent studies tracked the rise of the Negev viticulture as concomitant with involvement in circum-Mediterranean trade networks linking the Mideastern regions with Europe and noted a significant drop-off in commercial-scale, export-bound winemaking from the mid-sixth century CE (9, 11). Despite the Early Islamic (seventh to tenth centuries) and the Mamluk (13th century) enforcement of Muslim law that forbids wine production and consumption, vines continued to be cultivated for the local consumption of table grapes, raisins, and, in limited amounts, ceremonial wine among Jews and Christians (12, 13). However, knowledge of the specific grapevine cultivated in the Negev was lost.

While the modern Israeli wine industry relies on the noble 11 varieties (14), the region also contains feral grapevine landraces, which presumably descent from those used to produce the historic wines depicted in Judeo-Christian literature and theology. Over the past 150 y, dozens of indigenous Southern Levantine cultivars have been collected, studied, and described (15–17). Based on analyses of microsatellite allele frequencies (16, 18) and genomic sequences (19), the endemic cultivars were found to form a genetically distinct group. Today, specialized wineries and monasteries are producing small batches of wines using these cultivars. Yet, it remains unknown if these purportedly genuine varieties are in fact the product of age-old continuous local cultivation and are genetically linked with the ancient cultivars of the region.

Recent advances in paleogenomic techniques are transforming our understanding of past cultivar diversity by providing valuable datasets for the study of heirloom plants in Antiquity (20, 21). For example, ancient DNA (aDNA) extracted from French grape pips showed that the pedigree of some Western European heritage cultivars is rooted in medieval and Roman times (22). Remarkably, one 900-y-old specimen was identified as the genetic clone of *Savagnin Blanc*, a popular French cultivar, offering evidence of centuries of uninterrupted clonal propagation.

Here, we report the results of target-enriched genome-wide sequencing on DNA extracted from ancient grape pips found in a sealed stone-built room in the ancient settlement of Avdat (Oboda) in the Negev Highlands (23). The architectural style in the compound is Byzantine and affronts a cave dwelling space featuring Early Christian monastic wall paintings. Above the Byzantine period floor, thick accumulations of desiccated dung with abundant uncharred grape pips were dated to the Early Islamic period (*SI Appendix*, Table S1). To expand our research, we also sequenced modern indigenous cultivars and feral and wild grapes collected from across Israel. Comparative analyses of the modern and ancient datasets and of sequenced cultivars from around the world provided insights into the genetic legacy of the Late Antiquity grapes. Moreover, we discovered that one of the ancient specimens can be linked with an excellent winemaking grape lineage whose progenies are still cultivated today.

## Results

### DNA of Archaeological and Modern Native Samples.

**Fig. 1.**
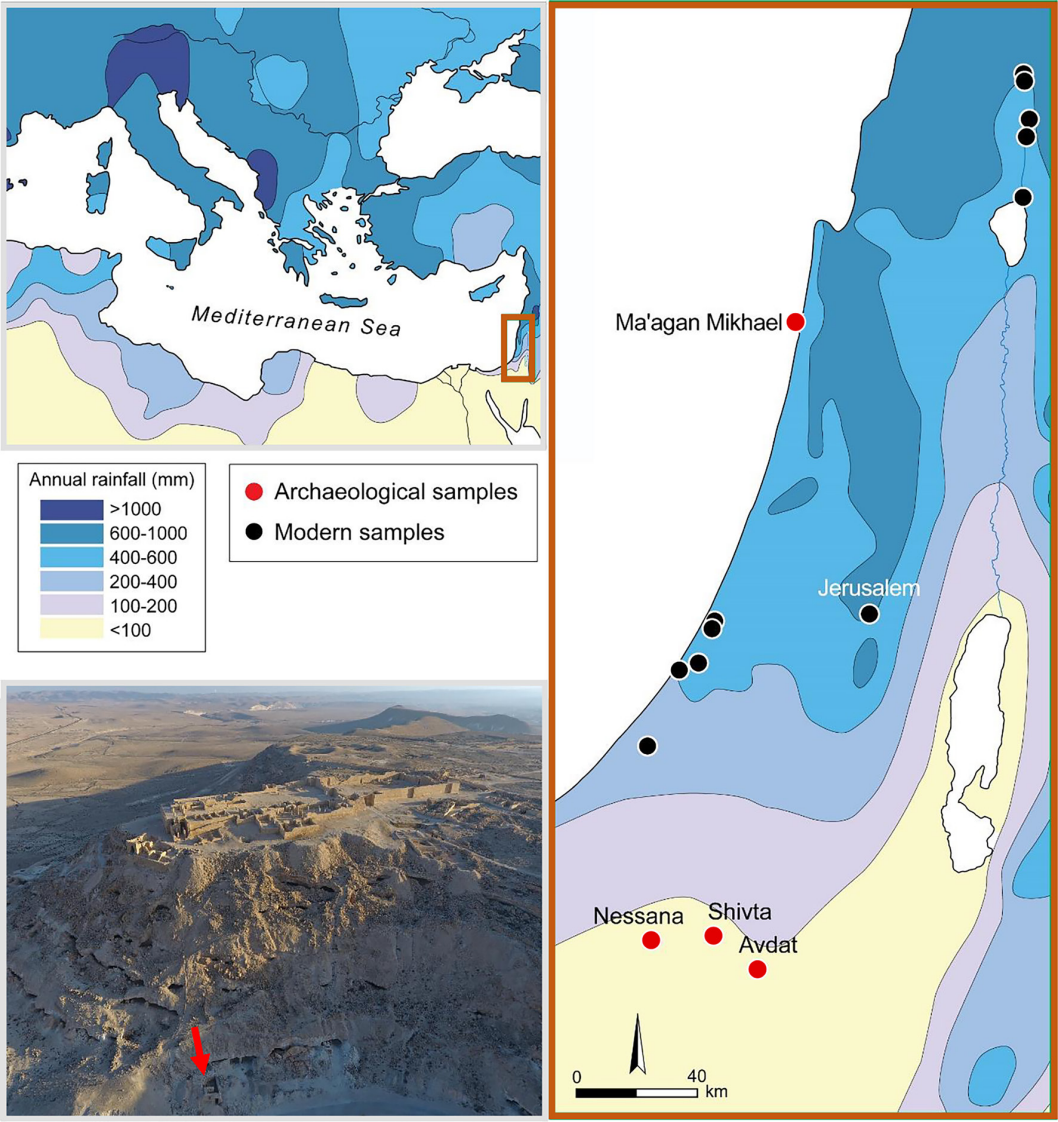
*Top* and *Right*. Geographic locations of the ancient and modern samples that were processed for this study, including annual rainfall data of the region, that is based on refs. (26, 27) and https://slideplayer.com/slide/4456125/. *Bottom Left*. An aerial view of the archaeological site Avdat. The arrow points to the recovery location of the five archaeological samples that were used in the analyses.

We extracted 16 archaeological grapevine pips from four archaeological sites in Israel (Fig. 1 and *SI Appendix*, *Chapter* 1 and Tables S1 and S4). Six pips successfully yielded DNA and were halved. One half was used for DNA extraction and the other half for radiocarbon dating. The radiocarbon dates ranged from the end of the seventh to tenth centuries CE (*SI Appendix*, Table S1). Eventually, five pips were used in this study. Nucleotide misincorporation patterns (24, 25) and read length distributions observed in the sequenced data were consistent with those expected of degraded DNA.

We also sampled and sequenced the young leaf tissues of 33 wild and domesticated grapevine varieties from across Israel. Eventually, nine unique samples were used in this study (Fig. 1 and *SI Appendix*, Tables S2 and S5). The ancient and modern samples were target enriched for 10,000 single-nucleotide polymorphisms (SNPs) (28) and then sequenced. We added the genotypes of hundreds of accessions from the GrapeRefSeq diversity panels (28, 29) and other publicly available grape genomes from several National Center for Biotechnology Information (NCBI) projects (19, 30, 31) (*SI Appendix*, Table S3).

Due to the high variability in quality among the ancient samples, we decided to create two separate datasets that either maximized the number of SNPs in the analyses (6,928 SNPs and three ancient samples; coverage X10-X40) or maximized the number of the archaeological samples (1,032 SNPs and five ancient samples; coverage X4.6 –X59). See *SI Appendix*, Tables S1 and S4 for more details.

### Archaeological Samples Fit within the Genetic Cluster of Southern Levantine Cultivars.

A principal component analysis (PCA) of 934 grapevine accessions from Europe, Western and Central Asia, and North Africa shows that the variation explained by the first principal component can mostly be attributed to differences between the wild and cultivated samples (*SI Appendix*, Fig. S4). This is consistent with the findings of prior studies (30, 32).

**Fig. 2.**
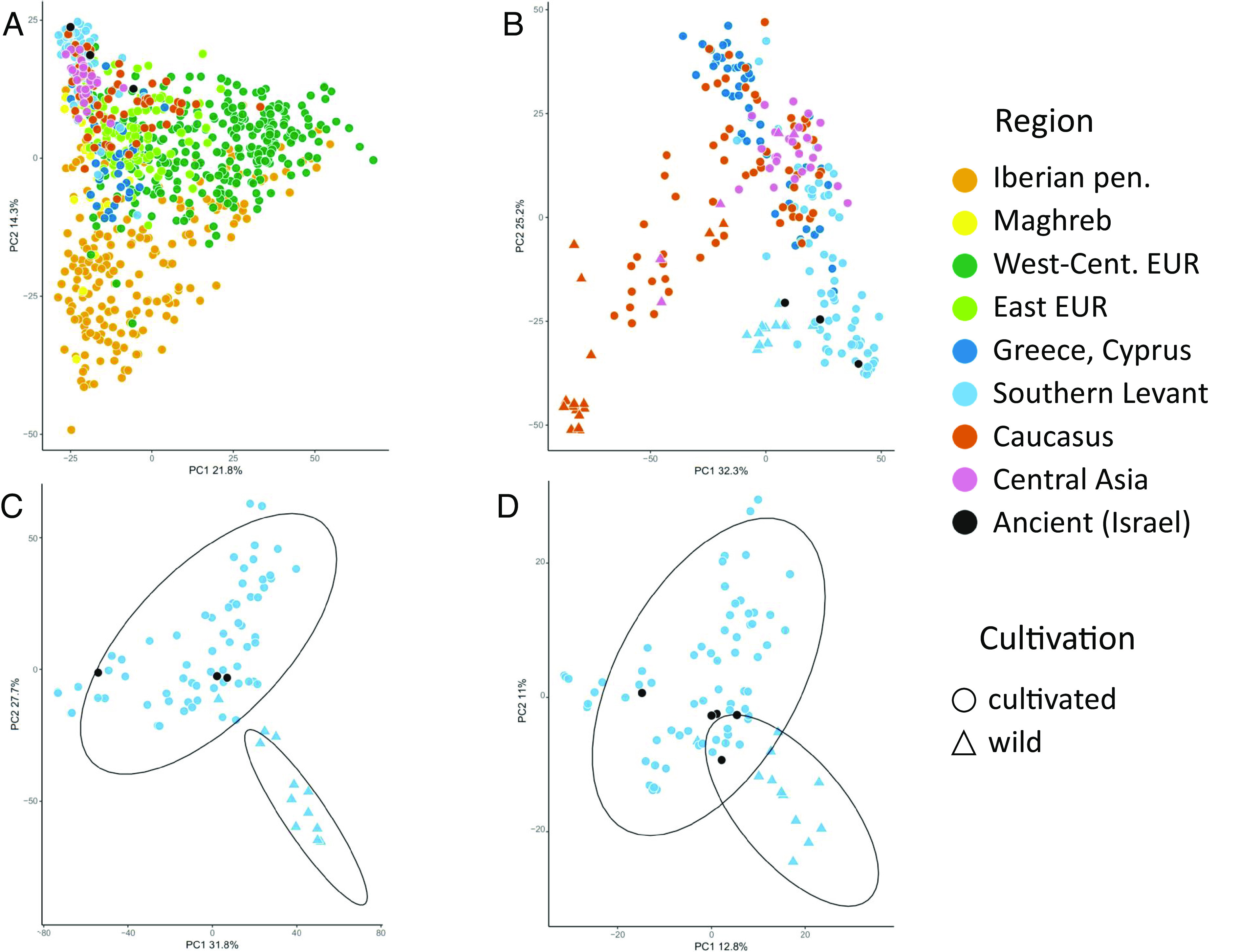
PCA plots of grapevine accessions over varying geographical scopes. (*A*) Cultivated European, Asian, and North African accessions. (*B*) Cultivated and wild Greek, Cypriot, Southern Levantine, and Asian accessions. (*C* and *D*) Cultivated and wild Southern Levantine accessions. *A*–*C* were plotted over the larger genotype datasets with only three archaeological samples and *D* over the smaller genotype datasets with all five archaeological samples.

When only the cultivated accessions were analyzed, a triangular genetic structure was revealed (Fig. 2*A*). One edge of the triangle outlines the axis from Western and Central Europe to the Iberian Peninsula. Another edge outlines the axis from Western Europe through Eastern Europe to Asia and the Levant. Interestingly, samples from the Southern Levant (Israel, Syria, and Lebanon in our dataset) were mostly clustered tightly together and appeared separate from other Asian locals. We repeated our analysis of cultivated and wild Eastern accessions only (the Asian and Greek groups) and found that the distinction between the Southern Levant samples is maintained and that the archaeological samples from Israel fit within the cluster of the Southern Levantine samples (Fig. 2*B*).

To determine whether the ancient samples were likely cultivated variety, we executed two PCAs over the Southern Levant samples using both SNP datasets. In both analyses, the archaeological samples fell within the cultivated range (Figs. 2 *C* and *D*). Our result was further supported by a *STRUCTURE analysis* (33) in which the archaeological samples were assigned to the same clusters as the cultivated accessions (*SI Appendix*, Fig. S8).

### Kinship between Southern Levantine Accessions.

**Fig. 3.**
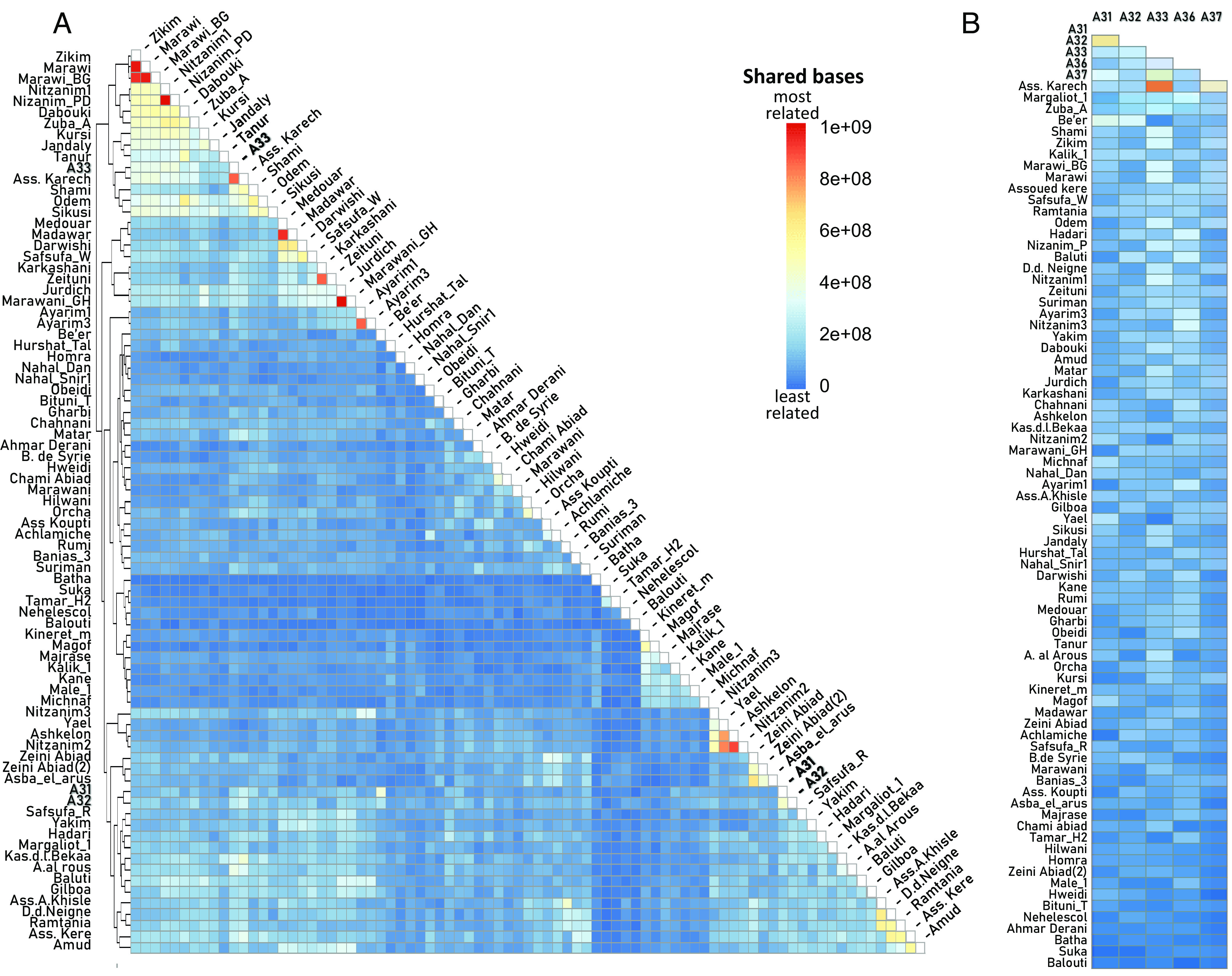
Kinship between archaeological (in bold) and modern Southern Levantine accessions based on the shared IBD segments between each sample pair. Variation in color from dark blue to dark red identifies relatedness based on the number of base pairs in shared IBD segments (more shared base pairs indicate closer relatives) (*A*) Kinship between all Southern Levantine accessions based on the larger SNP dataset. The samples are ordered by their hierarchical clustering. (*B*) Kinship with the archaeological samples based on the smaller SNP dataset. The modern accessions are ordered by their averaged closeness (based on total shared IBD segments) to the archaeological samples.

Fig. 3*A* shows the inference of kinship between all Southern Levantine accessions based on shared Identity by Decent (IBD) segments for the larger SNP datasets. Archaeological A33 and modern Asswad Karech cultivar, which was sampled in Lebanon, appear to be very closely related; their kinship appears stronger than that of known modern parent–offspring pairs (*SI Appendix*, *Chapter* 6 and Fig. S5). Archaeological A31 and A32 appear to be related to a lesser extent and so do A31 and modern Be'er, a feral variety growing wild along the southern Israeli coast.

Fig. 3*B* features the inference of kinship between the archaeological samples and the Southern Levantine accessions based on shared IBD segments for the smaller SNP datasets. It too shows A33 and Asswad Karech to be very close and gives support to a close kinship between A31 and A32 and between A31 and Be'er. In addition, archaeological A37 appears to be related to A33 and to Asswad Karech.

Further analysis of the accuracy of the imputation and phasing and how kinship is assessed are shown in *SI Appendix*, *Chapter* 6 and Figs. S5 and S6.

### Modern Asswad Karech Has Likely Parented an Ancient Grape.

We estimated kinship coefficient among pairs of Southern Levantine samples by running the program KING (34). Archaeological sample A33 and modern Asswad Karech were found to satisfy the parent–offspring criteria: Kinship coefficient (K) was estimated at 0.3017 and IBS0 at 0. IBS0 stands for the proportions of alleles in which neither chromosome is identical by state (i.e., the proportion of homozygote to homozygote mismatches). More details of the criteria of different relatedness categories are provided in *SI Appendix*, *Chapter* 8.

Allowing for 45 sequencing errors (for diploid 6,896 sites; minimal error rate 0.33%), all of A33 genotypes are contained in Asswad Karech, while the opposite is not true. We manually inspected the phased haplotypes and were able to match A33 haplotypes to Asswad Karech’s (*SI Appendix*, Fig. S7). This implies that A33 is descendent from Asswad Karech and from it alone either through selfing of one Asswad Karech plant or the breeding of two Asswad Karech clones. There are 1,976 heterozygous sites in Asswad Karech and only 913 in A33 (*SI Appendix*, *Chapter* 5 and Fig. S3). The roughly 1:2 heterozygosity ratio is on par with one or more generations of selfing/clonal breeding. For more details, see *SI Appendix*, *Chapter* 7.

No other close kinship relationship involving archaeological samples was found using KING. Two modern native Israeli samples were identified as clones (Ashkelon and Nitzanim [2]; K = 0.4798, IBS0 = 0). The complete results from KING analysis can be found in *SI Appendix*, Fig. S9.

### Berry Color of Archeological Samples.

**Fig. 4.**
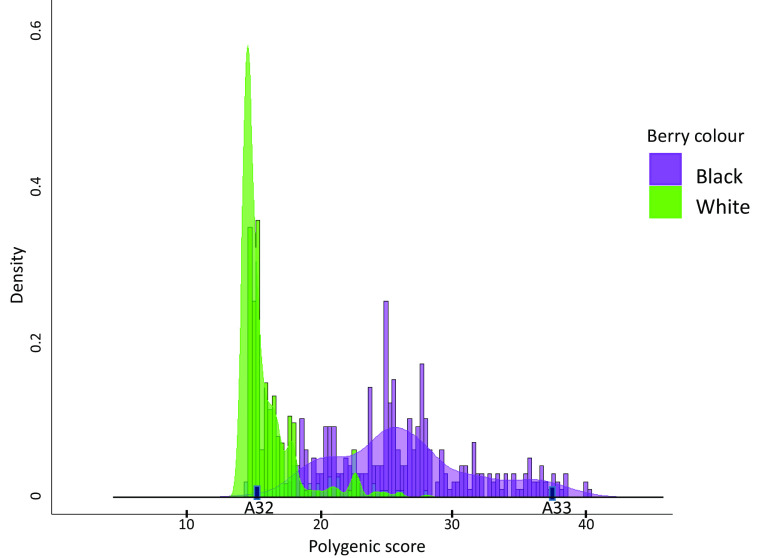
Histogram and density of the polygenic score of 19 genomic loci associated with color of 386 white and 332 black modern *Viti* accessions and of two archaeological samples, A32 and A33.

In a genome-wide association study (GWAS) of grapevines, 26 loci were found to be significantly associated with the quantitative trait of berry color (28). Of these loci, 19 had genotype calls with high coverage in archaeological samples A32 and A33. The combined genomic effect of the genotypes in these loci, known as the polygenic score, is used to estimate genetic predisposition of an individual to a certain trait. We calculated the polygenic scores of the genotypes of the two samples over these loci and compared them to the scores of 774 modern cultivated accessions with known berry color. The two archaeological samples fit in the two opposite extremes of the score distribution of the white and black cultivars (Fig. 4). Based on this limited number of loci, we can cautiously identify A32 as a light colored, probably white, grape. It scored lower than 97%, 90%, 82%, and 61% of the modern black, red, rosé, and white cultivars, respectively. A33 scored higher than all modern white, rosé, and red cultivars and of 94% of black cultivars, suggesting it is most likely a black grape. Due to their small sample sizes, the scores of the rosé and red modern accessions are presented separately in *SI Appendix*, Fig. S10.

## Discussion

Global warming has led to the critical search for grape varieties adapted to aridity. In this study, we focus on the arid Negev Highlands in Israel, examining pips which were excavated in Byzantine to Early Islamic sites. We applied radiocarbon, paleogenomic, and bioinformatic techniques on these pips and found that they were most closely related to traditional Southern Levantine cultivars. This result shows that the ancestry of Southern Levantine grapevine goes back at least 1,000 y, suggesting that the Eastern Mediterranean grape cultivars likely developed high levels of resilience to the harsh environment.

We inferred the parent–offspring relationship in the very least between a modern Lebanese cultivar, Asswad Karech, and one of the archaeological samples, A33. Most likely, A33 is a selfed Asswad Karech since the modern cultivar contains all of the haplotypes of the archaeological specimen but not the other way around. Asswad Karech is a black cultivar, characterized by big clusters of berries (35). In Greece, the same variety is known by the synonym Syriki and is mostly grown for table consumption and rarely for wine (35, 36). Its eastern origin is reflected in the name Syriki (either “from Syria” or “from the East”—“shark*”* in Arabic). However, this synonym may have a different interpretation. In biblical literature, *Sorek* is the name for a grape that was grown in the Judean Plain, an area located between the Judean Mountains and the coastal plain in contemporary Israel (37). Interestingly, in ancient Hebrew, “srak” means “red,” seemingly a direct reference to the color of Sorek berries.

An offspring of Asswad Karech is the Greek cultivar Ladikino (38), presumably named after the ancient Syrian port city of Laodicea (presently Latakia) (36) that was a Byzantine provincial capital until the Islamic conquest in the seventh century CE. The wine produced in Laodicea was exported across the Byzantine Empire and beyond, reaching Egypt, southern Arabia, East Africa, and India (39). Since the 11th century, Ladikino was cultivated in Crete, where it was used in the making of the famous Cretan *Malvasia* wine (36). The Malvasia brand name, under which several different wines were traded, is thought to be derived from Monemvasia (Malvasia in Italian) (40), a Byzantine fort and port city located in southern Greece. Our results are in line with the historical records and confirm the antiquity of Syriki and Ladikino.

Previously, some of the traditional cultivars of Greece, including Syriki and Ladikino, have been genetically and ampelographically linked with the Eastern Mediterranean (35, 36). A multitude of archaeological finds in Greece and Europe point to the popularity of wine from the Negev during the fourth to seventh centuries CE (10, 41, 42). We are unable to directly relate the sequenced Early Islamic Avdat grape pips to the Byzantine Negev wine industry. However, such a connection might be inferred from different sources. First, increasing evidence suggests that rainwater-harvesting agricultural infrastructure in the Negev, which peaked in the mid-Byzantine period, continued to be maintained into the Early Islamic period (43, 44). This is likely also true for the cultivars grown in Avdat. Furthermore, a study of viticulture intensity based on grape pip frequencies suggests that in the Early Islamic period, viticulture continued at monastic sites (9). Since the sequenced grape pips were found in an excavated monastic site at Avdat (45), it is plausible that they belonged to a variety cultivated in the Byzantine period.

Despite the small number of archaeological pips utilized in this study, three pips from the same locus (Spit S2, Locus 21), independently dated to similar periods, were found to be genetically diverse. Thus, it is possible that a variety of grape plants were cultivated together in the same vineyards and may have been used as a blend to produce wine. Another possible explanation for this is that the “multicropping” cultivation strategy was employed at Avdat. Diversifying crops diminishes risks and maximizes the utilization of field space and labor and was commonly practiced with grapes (13) among other crops in the ancient world (46, 47). This allowed the farmers to extend the harvest season and increase agricultural productivity by, for example, mixing early ripening varieties with late-ripening varieties. Innovations in water harvest and transport infrastructure changed the physical landscape, allowing the expansion of the Byzantine Mediterranean agricultural complexes into the previously uncultivated arid regions (48). These advanced technologies, possibly applied together with multicropping practices, have enabled farmers to thrive despite the harsh conditions of the arid Negev Highlands terroir. The extended study of the Avdat grapevine varieties enables a more complex comprehension of the methods past farmers used to bolster the landscape’s carrying capacity and to increase food security (49, 50).

The genetic diversity identified between the archaeological pips appears to include variations in berry color. Wild grapes generally have black berries, and this is the ancestral phenotype in grapes. Color variations in cultivated grapevine were found to be strongly associated with polymorphism in a cluster of genes of the VvMybA transcription factor family found on chromosome two of the grape genome (51, 52). Mutations in these regulatory genes lead to disruption in the production of the anthocyanin pigment which is responsible for the purple color in grapes and in other plants. While the precise shade is determined quantitatively, i.e., the reduction in the number of functional haplotypes progressively impairs the synthesis of anthocyanin and leads to lighter berries, normally only the complete absence of anthocyanin results in white grapes (53). This is reflected in the polygenic scores of the white cultivars presented in Fig. 4, where the bulk of the samples populates the lower end of the distribution. As the polygenic score of archeological sample A32 falls below those of most modern white grapes, it is likely that it did not express any anthocyanin.

Light color grape cultivars are today popular worldwide. Coalescence-based analysis of haplotypes associated with color variation showed that they carry the signature of a recent exponential expansion (51), which was likely driven by cultivators’ preference. It has been proposed that the ancestral haplotypes originate in the Iberian Peninsula and diffused eastward, accumulating additional mutations over time (54), while a second theory suggested a more Eastern origin (55). More recently, it has been shown that mutations disrupting the production of the anthocyanin protein can spontaneously occur; thus, the white color may have multiple origins (56). To date, archaeological pip A32 is the oldest specimen to be identified as a likely white grape.

The results of this study demonstrate the potential of ancient plants to provide unique insights into ancient agricultural practices. The well-preserved grape pips discovered in the Negev give evidence for the feasibility of outstanding molecular preservation even in warm climates. The genetic legacy of a Late Antiquity grapevine can now be explored through the winemaking grape lineage that demonstrates remarkable flexibility by being successfully cultivated in a range of environments (from desert to temperate). Future studies of the Asswad Karech cultivar may reveal the genomic loci responsible for resilience to aridity. Furthermore, in the current age of global warming, our results may support and guide selective grapevine breeding aimed at propagating cultivars with demonstrated ability to contend with the rising temperatures.

## Methods

### Archaeological Israeli Samples.

#### Sampling and radiocarbon dating.

Following Elbaum et al. (57), 16 well-preserved ancient grape pips were selected for DNA analyses and radiocarbon dating using Fourier transform infrared spectroscopy (*SI Appendix*, *Chapter* 2). The pips came from the Shivta, Nessana, and Avdat archaeological sites of southern Israel in the Negev Highlands and the Ma’agan Mikhael B shipwreck in the coastal plain of northern Israel (Fig. 1 and *SI Appendix*, *Chapter* 1 and Table S1). The ancient pips were split into two; one half was sent for DNA extraction, and when it was successful, the other half underwent radiocarbon dating at the Dangoor Research Accelerator Mass Spectrometry Laboratory in Rehovot, Israel. The radiocarbon dates were calibrated (to 2σ; *SI Appendix*, Fig. S1 and Table S1) in OxCal 4.4 (https://c14.arch.ox.ac.uk/oxcal/OxCal.html) using the IntCal 20 calibration curve (58).

#### DNA extraction, amplification, and sequencing.

The samples were processed in dedicated aDNA facilities at Tel Aviv University, University of Copenhagen, and University of York. The pips were grounded to fine powder using a pestle and mortar. DNA was extracted following the DNA extraction protocol of archaeobotanical remains of Wales et al. (2014) (59). More details in *SI Appendix*, *Chapter* 3.

DNA libraries were prepared for both shotgun (all archaeological samples) and capture (samples A31-A37) sequencing methods. For the shotgun sequencing, DNA extracts were converted into double-stranded Illumina sequencing libraries. Sample A31–A37 libraries were built using NEBnext DNA Library Prep Mast Mix Set 2 (E6070L, New England BioLabs) with modifications described by Wales et al. (60). Sample A140–A150 libraries were built using the blunt-end single-tube protocol (61). For the capture target sequencing, the libraries of samples A31–A37 were enriched for a set of 10,207 SNPs according to Ramos-Madrigal et al. (22) and Laucou et al. (28). Libraries were captured following the myBaits protocol version 3.0.

PCR was performed on a total volume of 100/25 μL:2/0.5 μL of a unique index oligo (10 μM) and primer IS4 (10 μM) (62) for capture and shotgun sequencing, respectively. DNA concentration was measured using the Qubit dsDNA high sensitivity (HS) Assay Kit (Invitrogen™) following the manufacturer’s protocol, and DNA was also quantified and visualized for length distribution using the High-Sensitivity D1000 DNA tapes on the TapeStation 4200 (Agilent Technologies). Finally, DNA libraries were pooled based on index compatibility and sample molarity. The libraries were sequenced on an Illumina 2500 HiSeq platform. More details about the sequencing procedure in *SI Appendix*, *Chapter* 3.

#### Processing of sequenced data.

PCR-duplicated reads were removed using a custom script, so only unique sequences remained. Adaptor contamination was removed using LeeHom software (63) and low-quality reads and reads shorter than 25 bp were removed using Trimmomatic version 0.36 (64).

The archaeological samples were mapped to the grapevine reference genome assembly (12X.v2) (65) using Bowtie2 version 2.2.5 (66). The mapped reads were evaluated for aDNA damage patterns (24) using mapDamage version 2.0 (25) to assert that the samples are indeed ancient (*SI Appendix*, Fig. S2). We also used mapDamage to rescale the ancient sequence bases quality and improve mapping and reducing the number of genotype call errors stemming from aDNA type damage. See detailed description in *SI Appendix*, *Chapter* 4 and Tables S6 and S7, on measures taken to avoid misdiagnosing deamination damage as polymorphism. We also excluded reads that aligned more than once with the reference genome with their second-best alignment having less than twice the number of mismatches as the best alignment. After this stage, five ancient samples were eliminated due to a low number of remaining reads (<20,000), leaving nine samples.

### Modern Native Israeli Samples.

#### Sampling.

A total of 33 young leaf tissues of indigenous grapevines were sampled for this study. Samples of native domestic varieties were collected from the Sataf Rescue Garden near Jerusalem and from plants growing feral along the southern coast of Israel (16). Samples believed to be native wild varieties were collected from the north of Israel (14) (Fig. 1 and *SI Appendix*, Table S2).

#### DNA extraction, amplification, and sequencing.

Young leaf tissues were processed in modern DNA laboratory facilities in Tel Aviv University and at the UMR AGAP Genotyping Platform (Montpellier, France). Using a pestle and mortar, the samples were grounded into fine powder while under liquid nitrogen. DNA was extracted using the DNeasy Plant Kit (QIAGEN Inc.) following the manufacturer’s protocol. Through microsatellites (simple sequence repeats) comparison with the reference grape Refseq panel (28), 15 clones of panel cultivars and between the samples themselves were identified, and eventually, only 18 unique samples were sequenced. Illumina libraries were captured at the UMR AGAP Genotyping Platform following the myBaits protocol presented by Ramos-Madrigal et al. (22). The Illumina sequencing was carried out by the GeT-PlaGe facility (INRAE, Genotoul, France).

#### Processing of sequenced data.

Raw reads were demultiplexed using the Je program version 2.0.2 (67); duplicated reads were removed, and low-quality reads and adaptor contaminations were trimmed or removed using Trimmomatic version 0.36, leaving sequences that were at least 75 bp long. Next, the reads were aligned to the grapevine reference genome using Bowtie2. Reads that were aligned with more than two mismatches and one gap opening were filtered out. We also excluded multiple aligned reads as described above. After this stage, five samples were eliminated due to a low number of reads remaining (<20,000), leaving 13 samples.

### Publicly Available Whole-Genome Sequences.

Publicly available whole-genome *Vitis* sequences were downloaded from the NCBI database. We obtained a total of 126 samples from the short-read archive bioproject accessions PRJNA647155 (19), PRJNA388292 (30), and PRJNA393611 (31), selected based on their sequencing quality (*SI Appendix*, Table S3), and for the last bioproject, based on name compliance with the Vitis International Variety Catalogue (VIVC) grape catalog and with Lacombe et al. (68). The samples were processed as described above for the modern native Israeli samples.

### Building of SNP Datasets.

SNPs calling for modern and ancient samples was done using HaplotypeCaller and GenotypeGVCFs protocols of the GATK pipeline (69). Overall, 15,414,158 SNPs were identified. About 9,988 of them were included in the 10K SNP array for which the captured samples were enriched. The remaining three shotgun ancient samples, one captured ancient sample, and nine of the native samples with coverage of less than one in this SNP array were eliminated, leaving only five ancient samples, all from Avdat, and nine native samples (*SI Appendix*, Tables S1 and S2).

Next, the genotypes of 783 cultivated and 112 wild grapevine samples from the GrapeReSeq diversity panels described by Laucou et al. (28) and by Le Paslier et al. (29), respectively, were incorporated in the genotype catalog. From this catalog, we created two separate SNP datasets to be used in the analyses: One included three ancient samples and 6,928 SNPs and the other all five ancient samples and 1,032 SNPs. For each dataset, we used VCFtools (70) to assert that each locus had genotype call in at least 75% of the samples, and each of the samples had genotype calls in at least 60% of the loci with the minimal read depth of five (for nonpanel samples). In all of the following analyses, the abovementioned quality criteria were maintained or made stricter.

### PCA.

We executed a PCA using the decomposition of the covariance matrix approach by running prcomp function implemented in R with the default parameters and scale=TRUE when possible. The results were plotted using the R packages factoextra and ggplot2. The samples were geographically classified according to Bacilieri et al. (55) with a few modifications and included only accessions sampled in Europe, Western and Central Asia, and North Africa. The full list of countries and their geographical groups can be found in *SI Appendix*, *Chapter* 8.

In the PCA that included only the Southern Levantine samples, the R function stat_ellipse was used to calculate and draw ellipses with 95% confidence level around the cultivated and wild samples assuming a multivariate t distribution.

### Kinship Analysis.

We inferred kinship between the Southern Levantine accessions in two different ways:

#### Genotypes phasing and inference of IBD segments.

Utilizing all available accessions, we only used loci with a minor allele frequency (MAF) of 3% that had genotype calls in at least 80% of the samples, leaving 6,896 and 1,030 SNPs, respective of dataset. The genetic data were imputed and phased to infer the lengths of shared IBD segments using Beagle and refined IBD software (71–73). Using this combined approach, IBD segments that are shared with one haplotype copy (IBD1) are built stochastically, and missing genotypes are imputed using the hidden Markov chain model. The logarithm of the odds (LOD) score of candidate segments is calculated based on the likelihood ratio in an IBD versus a non-IBD model. We were able to identify a total of 116,423 and 42,676 IBD haplotype segments with LOD > 3 among all pairs of Southern Levantine samples, respective of SNP dataset. We used the summed lengths of shared haplotypes between each sample pair to infer their relative relatedness. This analysis and the insepction of the phased haplotypes between A33 and Asswad Karech are the only analyses utilizing imputed SNP datasets. For the larger SNP dataset, the samples were ordered and clustered using the R library pheatmap.

#### Inference of kinship coefficient based on allele frequencies.

We ran the “KING-robust” algorithm implemented KING software version 2.2.7 (34) to estimate kinship coefficient between each sample pair based on genotype calls. This algorithm was chosen because it accounts for biases in kinship coefficient estimation which stem from population structure. The level of relatedness was assessed based on the resulting kinship coefficient (K) and IBS0 proportion and according to the relatedness categories shown by Laucou et al. (28) to be reliable for kinship inference in grapes (*SI Appendix*, *Chapter* 8). In this analysis, we used the larger SNP dataset.

### Berry Skin Color Analysis.

We used the GWAS for berry color described by Laucou et al. (28). 26 genomic loci were found to be significantly associated with grape color variation (*P* value < 5E−06 after Bonferroni correction for multiple tests). The effect of each of their alleles was calculated based on the grading of the five color categories (white, gray, rose, red, and black ranging between zero and four, respectively). Of these loci, 19 had genotype calls with the averaged coverage depth of X49 (X7–X206) in two archaeological samples, A32 and A33. The polygenic score for the color phenotype was calculated by summing the inferred allele effects over the genotypes in these loci. The polygenic scores of 774 modern *Vitis* accessions with known color (386 white, 27 rosé, 29 red, and 332 black), all with genotypes in all 19 loci, were also calculated.

## Supplementary Material

Appendix 01 (PDF)

## Data Availability

Genomic data have been deposited in NCBI (PRJNA887737 and PRJNA887039). Previously published data were used for this work [(28) (https://urgi.versailles.inra.fr/Species/Vitis/Data-Sequences/Genotyping-data) (19, 29 (NCBI project PRJNA647155) (30) (NCBI project PRJNA388292) (31) (NCBI project PRJNA393611 and PRJNA393611)].
