# Supplementary material for: Ancient DNA from a lost Negev Highlands desert grape reveals a Late Antiquity wine lineage
Source: Proc Natl Acad Sci U S A. 2023 Apr 17;120(17):e2213563120. doi: 10.1073/pnas.2213563120 (PMC10151551; doi:10.1073/pnas.2213563120)
Supplement: Supplementary file 1 — Appendix 01 (PDF) [file pnas.2213563120.sapp.pdf]

Supporting information for

Ancient DNA from a lost Negev Highlands desert grape reveals a Late Antiquity wine lineage

Cohen PninaΔ[1], Bacilieri Roberto[2], Ramos-Madrigal Jazmín[3], Privman Eyal[4], Boaretto Elisabetta[5], Weber Audrey[2], Fuks Daniel[6], Weiss Ehud[7], Erickson-Gini Tali[8], Bucking Scott[9], Tepper Yotam[10], Cvikel Deborah[11], Schmidt Joshua[10], Gilbert M. Thomas P.[12,3], Wales Nathan[13], Bar-Oz GuyΔ*[14], Meiri MeiravΔ*[1]

1. The Steinhardt Museum of Natural History and Israel National Center for Biodiversity Studies, Tel Aviv University, Tel Aviv 6997801, Israel
2. UMR AGAP Institut, Univ Montpellier, CIRAD, INRAE, Institut Agro, F-34398 Montpellier, France
3. Center for Evolutionary Hologenomics, The Globe Institute, University of Copenhagen, 1353 Copenhagen, Denmark
4. Department of Evolutionary and Environmental Biology, Institute of Evolution, University of Haifa, Haifa, Israel
5. Max Planck-Weizmann Center for Integrative Archaeology and Anthropology, DANGOOR Research Accelerator Mass Spectrometry Laboratory, Weizmann Institute of Science, Rehovot 7610001, Israel.
6. McDonald Institute for Archaeological Research Department of Archaeology Downing Street, Cambridge CB2 3ER
7. The Martin (Szusz) Department of Land of Israel Studies and Archaeology, Bar-Ilan University, 52900 Ramat Gan, Israel
8. Israel Antiquities Authority, 61012 Tel Aviv, Israel
9. Department of History, DePaul University, 2320 North Kenmore Avenue, Chicago, Illinois 60614
10. Zinman Institute of Archaeology, University of Haifa, Haifa, 3498837 Mount Carmel, Israel
11. Department of Maritime Civilizations and the Leon Recanati Institute for Maritime Studies, University of Haifa, Haifa, 3498838, Israel
12. Norwegian University of Science and Technology, University Museum, 7012 Trondheim, Norway
13. Department of Archaeology, University of York, York YO1 7EP, United Kingdom
14. School of Archaeology and Maritime Cultures, University of Haifa, Haifa, 3498837 Mount Carmel, Israel

*Equal contribution

ΔCorresponding authors. Emails: pninasmail@gmail.com; meirav.meiri@gmail.com; guybar@research.haifa.ac.il

This pdf file includes

Supporting text

Chapter 1: Archaeological sites information

Chapter 2: Pre-screening of the ancient grape pips

Chapter 3: Sample dating and ancient DNA sequencing in detail

- Samples dating
- DNA extraction, amplification and sequencing
    - Shotgun sequencing
    - Capture target sequencing

Chapter 4: Processing of archaeological samples in detail, handling of aDNA damage

- Processing of raw reads
- Mapping
- Mapped sequences filtering
- Genotyping and creating separate datasets
- Substitution frequencies analysis

Chapter 5: Archaeological samples homozygosity

Chapter 6: Robustness of inference of kinship through shared IBD

- Positive controls with known grape pedigree
- Control for error introduced through inaccurate imputation
- Control for error introduced through low coverage
- Accounting for ascertainment bias in the archaeological data

Chapter 7: A33 and Asswad Karech kinship

- A33 is likely the product of Asswad Karech selfing
- Shared haplotypes between A33 and Asswad Karech

Chapter 8: Additional analyses

- PCA of all samples
- STRUCTURE analysis of Levant samples
- Inference of kinship using KING

Supplementary tables S1 to S7

Supplementary figures S1 to S10

Chapter 1. Archaeological site information

The studied assemblages

The grape pips for this study were retrieved from three Byzantine towns, Shivta, Nessana and Avdat, which were part of a group of seven known urban centres from this period in the Negev Desert. The archaeological assemblages were retrieved from the multiple public buildings in these towns, including several churches and a basilica. In addition, grape pips from the Byzantine shipwreck Ma'agan Mikhael B, found off the coast of northern Israel, were also included in this study. Despite of decades of field work and theorization regarding the Negev Byzantine town phenomenon, only fairly limited sections of the towns underwent systematic and fully published excavations. The applications of bio-archaeological approaches were only carried out in the last several years (1).

Our recent excavations focused primarily on middens or trash mounds that provide an extended record of the growth and decline of urban settlements in the Negev. Compositional and mineralogical analyses of trash mound sediments, material finds and biological remains show that the mounds are composed of dense accumulations of both household and industrial trash (2–4). Grape seeds recovered in great abundance and ubiquity provide direct evidence for the extensive consumption of grape products and viticulture in the Negev (3). Ongoing archaeobotanical studies of Early Islamic contexts at Avdat give evidence of continuous grape growing and sophisticated wine production (6).

Shivta. The site reveals signs of abandonment as evidenced in the distribution and intentional barricading of doors at the Byzantine period household entrances (7, 8). Test-pit excavation in trenches within residential and public structures, water reservoirs, drainage channels, street and trash deposits show that the settlement was at its peak in

the Byzantine period (5th-6th c. CE) (8). Reduced intensity of human occupation is evidenced in Early Islamic period when trash began to be deposited inside abandoned houses. This phenomenon was documented as late as the 8th or 9th c. CE.

Nessana. The site presents a somewhat different story as it is located at a rather isolated position, yet a strategic one, along trade and pilgrimage routes, on the very edge of the Negev settlement system. Excavations of several trash middens in and outside the village show that occupation appears to have been sustained across the Byzantine-Early Islamic transition as do the Nessana papyri from the 7th century CE without much disruption. Small-scale removal of trash to exterior mounds continued into the Early Islamic period, while piles of trash appearing within structures on the periphery of the settlement date to as late as the 9th century (9).

Avdat. Since 2012, the Avdat in Late Antiquity Project has conducted fieldwork at the UNESCO World Heritage Site of Avdat (also known as Oboda or Abde) in the Negev Highlands of southern Israel (10). Excavation probes of Roman-period cave located on the southern slope of Avdat provide further evidence for continuity in settlement occupation, including wine growing and production. Red dipiniti drawings on the cave walls indicate that the cave and its external rooms were re-inhabited by a monastic community in the middle or late Byzantine period (10), which was corroborated by radiocarbon dating of a straw extracted mortar in the dipinti-intensive cave vestibule wall(6). Excavations conducted in 2016 and 2018 also revealed that the structure was used as a stable in the Byzantine and Early Islamic periods (5th – 9th centuries CE; (6, 10)).

In 2016, an excavation was carried out in front of the cave in a room with well-preserved walls making this one of the best-preserved external structures outside any of the caves in the site. The room is 2.60 × 3.40 m. in size and the southern and western walls were preserved to a height of over 2.8 m. and 3.1 m., respectively. The interior of the room

was covered with a layer of collapsed stone ceiling slabs that sealed the lower levels. The slabs, together with the location of the structure on the southern slope where it was less exposed to winter rains and run-off, ensured unusually well-preserved layers of rich organic material below the slabs.

A round, carved stone animal trough containing an outlet was revealed in the northwest corner of the room while a second stone carved installation, apparently used to hold a torpedo-shaped Gaza wine jar, was uncovered at a slightly higher level next to a doorway. Rich amounts of sediment rich in organic debris and animal dung were discovered in the southwestern corner of the room in Locus F5. Initially a mass of stems belonging to wildflowers were revealed here. Thereafter, the layers of debris were excavated in spits and carefully sifted under the direction of archaeobotanist, D. Fuks (Bar Ilan University). Well-preserved grape pips were discovered in some of the excavated spits, including the grape pips in Basket 21, Spit 2 and the pips in Basket 27, Spit 7, analysed in this study. Radiocarbon analysis of finds from the spits provided a range of dates in the Umayyad and Abbasid periods, between 650 and 890 CE ((6),Fig. 4). These data sheds new light on activity in the site in the post-Byzantine period and a tradition of viticulture there extending from as early as the fourth century CE.

Magan Michael B. The 23-m-long shipwreck was found 1.5m below sea level, 70m off the Mediterranean coast of northern Israel. The boat is in good state of preservation. Based on the radiocarbon dating of wood and organic finds and the typology of the complete amphorae, the shipwreck was dated to the early Islamic period (end of the 7th–beginning of the 8th centuries CE; (11, 12)). The most significant finds are the large quantities of well-preserved botanical remains, including olive pits, walnuts, peach stones, carob pods, pine cones and grape pips.

Chapter 2. Pre-screening of the ancient grape pips

Following Elbaum et al. (2005), we used Fourier Transform Infrared (FTIR) to estimate the preservation state of the grape pips, and to choose the best-preserved ones for DNA analysis. We powdered 0.1mg of the pip samples and mixed it with about 80 mg of spectroscopic grade KBr to produced pellets. The pellets were used to run the FTIR and the spectrograms were obtained from an average of 30 spectra collected at 4 cm^{-1} resolutions.

We compared the spectra of the grape pips to Lignin, cellulose and charcoal. We chose samples whose spectra resemble to lignin and cellulose, and showed that they contain uncharred organic residue. Most of the grape pips from Shivta and Nessana were charred while the pips from Avadt were well preserved.

Chapter 3. Radiocarbon dating and ancient DNA sequencing in detail

Radiocarbon dating

Radiocarbon dating of six halves of grape pips was done at the Dangoor Research Accelerator Mass Spectrometry (D-REAMS) laboratory in Rehovot, Israel. The radiocarbon dates were calibrated (to 2σ; Table S1) in OxCal 4.4 (https://c14.arch.ox.ac.uk/oxcal/OxCal.html) using the IntCal 20 calibration curve(58).

The model, a sequence of two phases, indicates the possibility that spit 2 is later than spit 7. The total calibrated range for each spit covers about 150 years. There are two possible interpretations: The two spits could be very close in time, around 780 CE, without a time gap between the spits deposition, or they could be separate by about 200 years. A cautious interpretation is that these grape pips were deposited there at different times between the end of the 7th and the 10th centuries CE.

DNA extraction, amplification and sequencing

The archaeological samples were processed in three dedicated aDNA facilities: Tel Aviv University (DNA extractions and libraries building). University of Copenhagen (libraries building, capture target sequencing, and whole genome sequencing), and the University of York (libraries building, capture target sequencing).

DNA was extracted using a protocol for DNA extraction from archaeobotanical remains, following Wales *et al.* (2014)(13). In short, the sample powder was incubated at 55°C overnight with 1 mL of digestion buffer containing: 10 mM Tris-HCl (pH 8.0), 10 mM NaCl, 2% w/v SDS, 5 mM $CaCl_2$, 2.5 mM EDTA (pH 8.0), 40 mM dithiothreitol (DTT), and 0.2% Protraeinase K. Then, DNA was extracted using a phenol-chloroform extraction protocol with three rounds of phenol cleaning. Samples were then purified using Qiagen QIAquick PCR Purification Kit with modifications described in Dabney *et al.* (2013)(14). DNA extracts were measured using a Qubit dsDNA High Sensitivity Assay Kit

(Invitrogem™) following the manufacturer’s protocol. Every fourth or fifth sample was blank (no sample added).

Shotgun sequencing

DNA extracts from the archaeological seeds were converted into double-stranded Illumina sequencing libraries. Samples A31-A37 libraries were built using NEBnext DNA Library Prep Mast Mix Set 2 (E6070L, New England BioLabs) with modifications described in Wales *et al.* (2015)(15). Samples A140-A150 libraries were built using Blunt-End-Single-Tube (BEST) protocol(16). Indexing of the PCR was performed on a total volume of 100/25 µL: 2/0.5µl of a unique index oligo (10 µM) and primer IS4 (10 uM)(17) for capture and shotgun sequencing respectively using AmpliTaq Gold DNA polymerase (5U/µl) (Applied Biosystems). Twelve to twenty-five cycles of amplification were performed (95°C for 10 minutes; 12-25x 95°C for 30 seconds, 60°C for 60 seconds, and 72°C for 45 seconds; 72°C for 5 minutes). PCR products were purified with a Qiagen QIAquick PCR Purification Kit. DNA concentration was measured using the Qubit dsDNA HS Assay Kit (Invitrogem™) following the manufacturer’s protocol, and DNA was also quantified and visualized for length distribution using the High-Sensitivity D1000 DNA tapes on the TapeStation 4200 (Agilent Technologies), following the manufacturer’s instructions. The libraries were pooled based on index compatibility and sample molarity. Libraries for the archaeological samples were sequenced on an Illumina 2500 HiSeq platform.

Capture target sequencing

The libraries of samples A31-A37 were enriched for a set of 10,207 SNPs according to Ramos Madrigal *et al.* (2019)(18), which corresponds with the GrapeReSeq diversity panel (https://urgi.versailles.inra.fr/Species/Vitis/GrapeReSeq_Illumina_20K). Libraries were captured following the MYbaits protocol version 3.0. Captured libraries were

amplified with PCR with 15 µL of captured libraries, 2× KAPA HiFi HotStart ReadyMix, 2.5 ul PCR Primer IS5, and IS6(19). Fourteen cycles of amplification were performed (98°C for 2min; 14x 98°C for 20 sec, 60°C for 30 sec, and 72°C for 30 sec; 72°C for 5 minutes). Amplified libraries were purified with a Qiagen QIAquick PCR Purification Kit. The libraries concentration was measured using the Qubit dsDNA HS Assay Kit (Invitrogem™) following the manufacturer's protocol, and the captured libraries were also quantified and visualized using the High-Sensitivity D1000 DNA tapes on the TapeStation 4200 (Agilent Technologies), following manufacturer's instructions. Finally, captured libraries were pooled based on index compatibility and sample molarity. Captured Libraries were sequenced on an Illumina 2500 HiSeq platform.

Chapter 4. Processing of Archaeological samples in detail, handling of aDNA damage

Processing of raw reads

Captured sequence data was successfully obtained for six samples and whole genome sequence data was obtained for 14 samples, which included the six capture sequenced samples. A total of 308,167,376 reads of 81 bp each was obtained, with an average of 40,780,059 reads for each captured sample and 4,232,468 reads for each shot-gun sequenced sample. PCR duplicated reads were identified based on sequence content using own perl script and were removed, leaving an average of 22,994,320 and 3,522,278 reads per sample for capture sequenced and shotgun sequenced samples respectively. See Table S4.

Ancient DNA sequences are generally very short, therefore we used LeeHom(20), a trimming program that optimizes adaptor trimming for very short reads. Further removal of low quality sequences was done using Trimmomatic version 0.36(21), using a sliding window of four bp requiring the Phred score of at least 15 and filtering out sequences that were less than 25bp long, in accordance with Poullet & Orlando (2020)(22).

Mapping

In order to eliminate substitutions that are the result of deamination damage to the DNA and were not real polymorphism, sequences of ancient samples were mapped to the reference genome of *V. vinifera* (12XV2) in two rounds. In the first round, Bowtie2 was run with the parameters 'end-to-end' and 'very-sensitive' to produce bam files that were used as input to the MapDamage software. No filtering took place at this stage. MapDamage produces Bayesian estimates to the cytosine deamination rates in the sequences (C->T and G->A substitutions), see Fig. S2. In addition, it re-evaluates the sequencing quality scores for each base pair (rescaling) according to the probably of it

being damaged due to deamination. In the second mapping round, the rescaled sequences which resulted from MapDamage analysis were inputted again to Bowtie2 with the same parameters as described above. The rescaled scores helped to reduce the number of incorrect mapping due to deamination damage. See Table S6, and detailed description in 'Substitution frequencies analysis' at the end of this chapter. In addition, through the second run of Bowtie2, confidence in the quality of the mismatching codons was reduced and so were the sequences mapping quality scores (Qmap) in comparison of the first run. These were used in the filtering round (next).

Mapped sequences filtering

The mapped reads were filtered based on the quality of the mapping, allowing a minimal Qmap of 8. Additionally, mapped reads with more than eight mismatches were filtered out. We allowed a relatively high number of mismatches so that sequences with ancient DNA type damage may still be used for genotyping, while confidence in their sequencing accuracy is reduced through the change in their sequencing quality field. Reads that were multiply aligned were also removed. Multiply aligned reads were defined as reads that were aligned more than once with the reference genome and their second best alignment had less than twice the number of mismatches as the best alignment. An average of 6.8% of the reads in the capture sequenced samples and 4.1% of the reads in shotgun sequenced samples remained at this stage.

After this stage, five samples were eliminated due to a low number of remaining reads (<20,000), leaving nine samples. The shotgun and captured sequences of the six samples that were sequenced in both methods were unified.

Genotyping and creating separate datasets

After genotyping using standard GATK pipeline(23) and the HaplotypeCaller and GenotypeGVCFs protocols. 9,988 SNPs were included in the 10K SNPs array for which

the captured samples were enriched. The remaining three shotgun ancient samples, one captured ancient sample and nine of the native samples had the coverage of X1 or less in this SNPs array were eliminated, leaving only five ancient samples, all from Avdat

Because of the high variability in quality among the remaining five archaeological samples as shown in Table S4, it was decided to create two separate datasets. One was used in analyses that required a large number of SNPs and initially included 6,939 SNPs in 116 accessions including three ancient samples. The other dataset is used in analyses that utilized all five ancient samples and initially included 1,033 SNPs in 118 accessions. The SNPs and samples were filtered so in each dataset all samples had genotype calls in at least 60% of the SNPs with a minimal read depth of five for the non-panel samples, and each locus had genotype call in at least 75% of the samples. These criteria were maintained or were made stricter throughout all of the analyses. For SNPs and samples filtering and for the statistical data required for filtering, we used *VCFtools* software version 0.1.13(24). To be able to include as many of the ancient samples as possible in the smaller SNPs dataset, we manually inspected and used additional custom scripts to choose the set of SNPs that fulfilled the cut-off of a minimum of 60% genotypes call in all five ancient samples.

The two alleles of each locus were checked against the known polymorphism described in Laucou *et al* (2018). Eleven SNPs in the larger dataset, and one in the smaller dataset were found not to have the expected alleles and were eliminated, resulting in datasets of 6,928 and 1032 SNPs respectively. For these SNPs, the ancient samples averaged in coverage between X4.6 and X58 (Table S1). The low error rate of 0.15% and lower (11/6,937 and 1/1,033), gives support for the chosen methods of ancient DNA type damage handling and the pipeline of processing, genotyping and filtering. In addition, the genotyping of the ancient samples together with the modern samples and the use of only known modern polymorphic sites helped in reducing erroneous genotype calls

due to ancient DNA damage. See detailed description 'Substitution frequencies analysis' at the end of this chapter. This practice also prevented the identification of any potential polymorphism which is specific to the ancient samples.

The archaeological samples averaged in coverage between X4.6 and X59 for these datasets and the modern native samples averaged in coverage between X72 and over X500, see Tables S1&S2.

Substitution frequencies analysis

We followed the changes in the numbers and in the frequencies of all of substitution types within the ancient samples (when compared to the reference genome) starting with the first mapping round and ending in the genotypes. This is summarized in Table S6. We found that the combined frequencies of C->T and G->A type substitutions are reduced from 47.6% of all substitutions in the first mapping stage, to 33.1% in the filtered genotypes stage.

For comparison, we calculated the same statistics over the nine modern Israeli native samples that were capture sequenced. This is summarized in Table S7. For these samples, the combined frequencies of C->T and G->A substitutions totalled in 28.8% in the mapped reads stage and in 32.6% in filtered genotypes. We also showed that the frequencies of all types of substitution are similar between the ancient and the modern samples when measured over the polymorphic sites that were eventually used (shown for the dataset of 6,928 SNPs). In particular, C->T and G->A substitutions are slightly reduced in the archaeological samples when compared with the modern samples.

Chapter 5. Archaeological samples homozygosity

On average, the archaeological samples have less than half of the number of heterogenic genotype calls as the modern samples (29.4% and 13.2% respectively, on our larger SNPs dataset). We initially checked weather loci in archaeological samples were misdiagnosed as homogenous due to insufficient coverage. We filtered the loci in the larger SNPs dataset to have the minimal reads depth (minDP) of 5 and the minDP of 10 and received similar homozygocity ratios (11.5%, 12.5% and 13.2 with minDP = 5 and 12.2%, 12.8%, 13.3% with minDP = 10 for archaeological samples A31, A32 and A33, respectively), and so we do not consider the evident ancient homozygosity as a consequence of insufficient coverage.

A possible explanation is by how the loci we were working on were chosen: they are polymorphic in modern cultivars, and so are used in identifying cultivars. Indeed, when the heterozygosity ratios of the cultivated and the wild accessions are inspected separately, there is a substantial difference (an average of 32.4 % for cultivated and 22.8% for wild). However, the archaeological samples are more homogenous than almost all of the wild accessions. See homozygosity distribution of cultivated and wild accessions compared with the ancient samples in Fig. S3.

Chapter 6. Robustness of inference of kinship through shared IBD segments

Innate aDNA problems of fragmentation, deamination and low coverage may affect imputation and the phasing analysis due to inaccurate genotyping of the archaeological samples. However, multiple studies have shown that the phasing and imputation of aDNA are useful(25–27) and may even be comparable in accuracy to the phasing and imputation of modern DNA when a large reference panel size is used(28, 29).

Other confounding factors can negatively influence the accuracy of the analysis. In our dataset such factors may include a non-homogenous SNPs distribution, ascertainment bias (see Chapter 5), the possible effect of grape plant selfing and of repeated breeding of some grape cultivars across generations and populations(30) on cultivated grape population structure.

Each of these issues are addressed bellow to show that our analyses and the conclusions drawn from them are robust to their effect.

Positive controls using known grape pedigree

Among the southern Levant samples there are two known clones (Karkashani - Zeituni and Marawi – Marawi_GB) and five known parent-offspring pairs (Asswad abou Khisle - Doight du neigne, Dabouki - Odem, Asswad Kere - Doight du neigne, Dabouki – Marawi and Dabouki – Marawi_GB)(30, 31). In the analysis of the larger SNPs dataset (81 samples; 3,240 possible pairs), these seven pairing combinations were found amongst the 33 most related pairs. In the analysis of the smaller SNPs dataset (83 samples; 3,403 possible pairs), these seven pairs were found among the 50 most related pairs. See Fig. S5.

Among the remaining high scoring pair combinations there are other apparent pairs of clones or close relatives. They include cultivar pairs whose names are spelt slightly

differently, such as Medouar - Madawar, or samples of unknown cultivar that were collected in the same areas such as Nitzanim_1 – Nitzanim_P.

We used the seven pairs with known kinship to assess the level of kinship with the archaeological samples. In addition, we use them as controls when testing for error introduced due to biases. See below.

Control for error introduced through inaccurate imputation

To determine if errors were introduced in the phasing analysis through erroneous imputation we rerun the phasing analysis over a SNPs dataset in which no missing data was allowed.

We filtered out 79 samples (out of the total 1,007) with more than 3% missing data and SNPs that had missing data in the remaining samples and were left with 5,146 SNPs with genotypes in 928 samples, of which 50 were southern Levant samples, including two archeological samples (A32 and A33). We repeated the phasing as described in the methods for this dataset and identified a total of 40,127 shared IBD segments (no missing data analysis). As control, we repeated phasing for the original dataset with the same 928 samples and randomly chosen 5,146 SNPs, and identified a total of 35,457 shared IBD segments (missing data analysis).

We present the lengths of shared IBD segments between each sample pair in Fig. S6A. All known clones and parent-offspring pairs rank similarly with and without missing data. A33-Asswad Karech pair also ranks similarly, thus the ranking is robust to error caused by erroneous phasing.

Control for error introduced through low coverage

To check for the effect of low coverage over the accuracy of the phasing analysis, we doubled the minimal number of reads required for each SNP (from 5 to 10), if possible.

Samples taken from Laucou 2018(30) are available only as genotypes and so we could not alter their coverage cutoff. We were left with 6,126 SNPs.

We filtered out samples with less than 60% genotype calls in 6,126 SNPs, and were left with 959 samples, of which 66 were southern Levant samples, including two archeological samples (A32 and A33). We repeated the phasing as described in the methods for this dataset and identified a total of 70,913 shared IBD segments (10X analysis). As control, we repeated phasing for the original dataset with the same 959 samples and randomly chosen 6,126 SNPs, and identified a total of 71,547shared IBD segments (5X analysis).

We present the lengths of shared IBD segments between each sample pair in Fig. S6B. All known clones and parent-offspring pairs rank similarly when the X10 and the X5 analyses are compared. A33-Asswad Karech pair also ranks similarly, thus the ranking is robust to error caused by low coverage.

Accounting for ascertainment bias in the archaeological data

Ascertainment bias in the archaeological data, such as the excess in homozygosity compared with the modern data, discussed in Chapter 5, may reduce the power of the analysis to correctly identify IBD segments in the archeological samples (i.e. false negative). To make sure there is no reason for concern for the wrong inference of kinship (false positive), we employed two additional analyses that supports the conclusion that A33 and Asswad Karech are highly related and apparently parent-offspring. One is the use of the program KING and the other is the manual inspection of the phased IBD segments between archeological A33 and modern *Asswad Karech*, described in Chapter 7.

Chapter 7. A33 and Asswad Karech kinship

A33 is likely the result of Asswad Karech selfing

KING analysis concluded with Asswad Karech and archaeological A33 satisfying parent-offspring kinship criteria. Importantly, there are no sites in which neither chromosome of these samples is not identical by state (i.e. the two samples can be matched with at least one of their chromosomal copies across the entire genome), which points to a close relationship, parent-offspring in the very least. We inspected the samples genotypes and found that Asswad Karech has approximately twice the number of heterozygote sites as A33 (1,976 and 913 respectively). In addition, there are only 45 sites in which Asswad Karech is homozygote and A33 is heterozygote. This is an exceptionally low number. For comparison, there are 1,108 sites in which the opposite is true and Asswad Karech is heterozygote and A33 is homozygote. In diploid species, if the parents are unrelated, heterozygosity ratio is not expected to change between generations, and a parent and an offspring are expected to share 50% of their haplotypes on average. Here, with the exception of these 45 loci, A33 carries no haplotypes that do not exist in Asswad Karech, while the opposite is not true. Assuming that all 45 sites are genotype errors, this strongly suggests that there was no genetic contributor to A33 other than Asswad Karech, which is in line with selfing or two clones breeding. On average, a 50% decrease in heterozygosity is also expected under such scenario. Together, these findings point to Asswad Karech as being the sole parent of A33. Any number of selfing generations is in line with the 1:2 heterozygosity ratio difference, as long as in each generation one parent is an original Asswad Karech.

Shared haplotypes between A33 and Asswad Karech

We carried out a manual inspection of the phased genotypes of A33 and Asswad Karech and were able to match all of the haplotypes of A33 with the haplotypes of Asswad

Karech, allowing for 113 mismatches (including the 45 mismatches mentioned above) and 30 crossovers. See Fig. S7. We chose to restrict the distance between two crossovers to at least 3% of the total length of the chromosome. Without this restriction, the additional 68 mismatches could have been resolved with more crossovers, and so these mismatches are not considered to be genotype errors.

While phasing allowed us to identify which of Asswad Karech haplotypes was probably inherited to A33, we do not suggest that the crossovers depicted in Fig. S7 accurately represent true recombination events that occurred during meiosis. The mismatches and the possible inaccuracies in the number and location of the inferred crossovers are probably the result of the phasing algorithm reaching its precision limits and to uncertainties in the manual inspection.

Chapter 8. Additional details for analyses

PCA of all samples

Composition of geographical groups by country is based on Bacilieri *et al.* (2013)(32), with the following changes: Greece and Cyprus were taken out of the Balkan group, which was also divided into two: the Balkan and East Europe. Russia, Ukraine and Moldova were included in East Europe. We also separated between the Levant (Eastern Mediterranean) and the Caucasus.

The full group list by country is: Iberian Peninsula: Spain and Portugal; Maghreb: Tunisia, Algeria, Morocco; West-Central Europe: Holland, Germany, Austria, France, Italy, Czech republic, Switzerland and Belgium; East-Europe: Bulgaria, Hungary, Romania, Slovenia, Bosnia and Herzegovina, Serbia and Croatia; Greece and Cyprus; Russia, Ukraine and Moldova; Levant: Israel, Syria and Lebanon; Caucasus: Armenia, Azerbaijan, Georgia and Turkey; Central Asia: Uzbekistan, Afghanistan, Iran, Tajikistan, Turkmenistan and Pakistan.

Population structure analysis

For the population structure analysis, we used the larger SNPs dataset with only the southern Levant samples and required a minimal minor allele frequency (MAF) of 3% in each genomic locus, which left 5,633 loci. We ran the program *STRUCTURE* over *K* (number of expected clusters) values of 2 – 7. For each *K* value, we ran *STRUCTURE* over 100 sets of 1,000 SNPs randomly chosen out of the SNPs collection. Multiple runs over subsets of the data contributed to the robustness of the clustering inference and allowed us to use more SNPs than would be possible in a single run. To avoid linkage disequilibrium, we required a distance of at least 3,000 bp between sites. We ran the program with 1,050,000 MCMC repetitions with the first 50,000 discarded (burn-in

period). All other parameters were kept at default values. The combined results of all together 600 *STRUCTURE* runs were graphically summarized using *CLUMPAK* (33).

Inference of kinship using KING

Laucou *et al.* (2018)(30) used genetic data of modern accessions with known pedigree to infer reliable kinship criteria for grapevine cultivar pairs from KING(34) run output parameters: K, the kinship coefficient and IBS0, the proportion of homozygote to homozygote mismatches:

1. Identical clones were defined as those with K > 0.354 and IBS0 <= 0.006 (allowing for the sequencing error of 1/1000 sequenced bases for the 6,896 loci used).

2. Parent–offspring relatives are defined as those with 0.177 < K < 0.354 and IBS0 <= 0.006.

3. Related are defined as all other identifiably related individuals for which 0.177 < K < 0.354 and IBS0 > 0.006 (full siblings according to the definitions in KING manual) and 0.177 > K > 0.0442 (2nd and 3rd degree relatives according to the definitions in KING manual).

For all other output parameter values, the sample pair in question are regarded as unrelated.

Tables

Table S1. Archaeological Israeli samples

Lab #	Site	Material	Excavati on date	Locus/spit	Basket/ sample	C14 lab number	C14[1]	±	from[2]	To[2]	%	**Used in analys es**	Coverage[3]	Coverage[4]
A31	Avdat, Israel	Half Seed	2016	S2	B21-3	RTD9067	1165	27	772	975	95.45	yes	X10	X18
A32	Avdat, Israel	Half Seed	2016	S2	B21-6	RTD9068	1126	29	774	995	95.45	yes	X40	X59
A33	Avdat, Israel	Half Seed	2016	S2	B21-7	RTD9069	1218	23	706	884	95.45	yes	X39	X56
A35	Avdat, Israel	Half Seed	2016	S7	B27-4	RTD9070	1349	26	644	774	95.45	no		
A36	Avdat, Israel	Half Seed	2016	S7	B27-6	RTD9071	1262	26	668	828	95.45	yes		X6.3
A37	Avdat, Israel	Half Seed	2016	S7	B27-11	RTD9072	1241	33	678	881	95.45	yes		X4.6
A140	Avdat, Israel	Half Seed	2016	8	d_3.1							no		
A141	Avdat, Israel	Half Seed	2016	8	d_3.2							no		
A142	Avdat, Israel	Half Seed	2016	8	d_3.8							no		
A143	Maagan Michael, Israel	Half Seed	2017	Amphora	MMB69 1							no		
A145	Maagan Michael, Israel	Half Seed	2017	Amphora	MMB69 2							no		
A146	Nessana, Israel	Half Seed	2016	601	6002.1							no		
A147	Nessana, Israel	Half Seed	2016	601	6002.1							no		
A148	Maagan Michael, Israel	Full Seed	2017	Amphora	MMB69 1							no		
A149	Maagan Michael, Israel	Full Seed	2017	Amphora	MMB69 2							no		
A150	Shivta, Israel	Half Raisin	2016	551	5509							no		

[1] Uncalibrated BP
[2] Calibrated CE
[3] Dataset 6,928 SNPs
[4] Dataset 1,032 SNPs

Table S2. Modern native Israeli samples

Sample	Coordinates (N, E)	Source	Localization	Cultivar	Used in the analyses (yes/no)	Coverage (dataset of 6,928 SNPs)	Coverage (dataset of 1,032 SNPs)
GB80	31.744003, 34.628468	Holot Nitzanim	Nitzanim (1)	Feral	yes	>X300	>X400
GB81	31.746405, 34.633308	Holot Nitzanim	Nitzanim (2)	Feral	yes	>X300	>X300
GB82	31.746403, 34.633617	Holot Nitzanim	Nitzanim (4)	Feral	no		
GB83	31.746521, 34.633904	Holot Nitzanim	Nitzanim (5)	Feral	no		
GB84	31.725834, 34.624443	Holot Nitzanim	Nitzanim (3)	Feral	yes	X142	X170
GB85	31.635765, 34.577345	Gilat	Ashkelon	Feral	yes	X72	X95
GB86	31.635281, 34.576921	Gilat	Ashkelon (4)	Feral	no		
GB87	31.629894, 34.577369	Gilat	Ashkelon (5)	Feral	no		
GB88	31.629967, 34.577360	Gilat	Ashkelon (6)	Feral	no		
GB89	31.738896, 34.622627	Gilat	Nitaznim2	Feral	no		
GB90	31.406517, 34.420081	Gilat	Nahal Nitzra	Feral	no		
GB91	31.632693, 34.580421	Gilat	Ashkelon (2)	Feral	no		
GB92	31.611768, 34.518628	Gilat	Zikim	Feral	yes	>X400	>X500
GB93	31.629945, 34.577369	Gilat	Ramat Divshon Merkaz	Feral	no		
GB94	31.771535, 35.127889	Sataf		Romi	no		
GB95	31.771535, 35.127889	Sataf		Baloti	no		
GB96	31.771535, 35.127889	Sataf		Jindaly	no		
GB97	31.771535, 35.127889	Sataf		Marawi Bet Jan	no		
GB98	31.771535, 35.127889	Sataf		Dabouki Cranialy	no		
GB99	31.771535, 35.127889	Sataf		Hilowani	no		
GB100	31.771535, 35.127889	Sataf		Dabouki Masarik	no		
GB101	31.771535, 35.127889	Sataf		Dabouki Arob	no		
GB102	31.771535, 35.127889	Sataf		Tufahi	no		
GB103	31.771535, 35.127889	Sataf		Beituni	no		
GB104	31.771535, 35.127889	Sataf		Zitoni	no		
GB105	31.771535, 35.127889	Sataf		Zani Hebron	no		
GB106	33.23396, 35.627666	North of Israel	Nahal Snir	Wild	yes	X67	X79
GB107	32.900875, 35.629143	North of Israel	Batha	Wild	yes	>X300	>X300
GB108	33.215649, 35.632553	North of Israel	Nahal Dan	Wild	yes	>X300	>X400
GB109	33.217457,35.629436	North of Israel	Hurshat Tal	Wild	yes	X73	X82
GB110	33.062813, 35.63501	North of Israel	Dardar-Betha	Wild	no		
GB111	33.109875, 35.645428	North of Israel	Golan heights, east of Gonen	Wild	no		
GB112	32. 897236, 35.629318	North of Israel	Zaki	Wild	no		

Table S3. Publicly available modern accessions used in this study

Source	Number	Type	Cultivation	Localization
Laucou *et al.* (2018)(30) (https://urgi.versailles.inra.fr/Species/Vitis/Data-Sequences/Genotyping-data)	783	Genotypes of 10,207 SNPs	783 cultivated	Europe, Asia, north Africa and USA
Le Pasliear *et al.* (2019)(35)	112	Genotypes of 9,896 SNPs (included in the 10,207 SNPs)	112 wild	Europe and Asia
Sivan *et al*, 2021(36) (NCBI project PRJNA647155)	47	Whole genome sequences	37 cultivated and 10 wild	43 Israelis, four Europeans
Zhou *et al* 2017(37) (NCBI project PRJNA388292)	21	Whole genome sequences	13 cultivated and 8 wild	Europe and Asia
Liang 2019(38) (NCBI project PRJNA393611)	58	Whole genome sequences	58 cultivated	Europe and Asia

Table S4. Number and frequencies of archaeological sequences reads in the processing and mapping stages. Colours are relative to amounts in the same column with green being higher number/percentage of reads, orange and yellow are midrange and red is low.

		Preprocessing				Mapping					
**Sample**	**Sequencing**	**Raw reads**	**removal dup**	**After trimming**	**% afer preproc**	**1st Mapped**	**2nd Mapped**	**Mapped%**	**Mapped-filtered**	**filtered %**	**% from raw**
A31	capture	12881619	6515554	6407403	49.740665	4238454	4228781	65.99836	2681773	63.41716	20.8186021
A32	capture	71606568	31122881	30933385	43.199089	8200143	8182307	26.45138	5305218	64.83768	7.40884272
A33	capture	60006648	33409219	33223595	55.366524	9189256	9169885	27.60052	5809777	63.35714	9.68188891
GV140	shotgun	915219	263116	253381	27.685286	3603	3574	1.410524	1785	49.94404	0.19503529
GV141	shotgun	1816510	457212	444830	24.488167	22279	22185	4.987299	13792	62.16813	0.75925814
GV142	shotgun	1101022	186012	182459	16.571785	912	908	0.497646	417	45.92511	0.0378739
GV146	shotgun	7168840	5845639	5770943	80.500374	432541	431597	7.478795	261680	60.63063	3.65024188
GV147	shotgun	603393	151672	146836	24.335052	7411	7384	5.02874	4601	62.3104	0.76252128
GV148	shotgun	7811071	5422810	5352287	68.521807	450785	449770	8.403324	255235	56.74789	3.26760568
GV149	shotgun	6814930	4918276	4857673	71.279866	1094212	1094213	22.52546	627984	57.39139	9.21482686
GV150	shotgun	6743730	5625751	5501694	81.582359	39886	39336	0.71498	15225	38.705	0.22576527
A31	shotgun	2020378	1813434	1728505	85.553545	824378	822242	47.56955	478153	58.15234	23.6665119
A32	shotgun	5757240	5681354	5643093	98.017331	810374	808348	14.32456	525008	64.94826	9.11909179
A33	shotgun	4749739	4638039	4609093	97.038869	678509	676709	14.68204	434293	64.17722	9.14351294
A35	capture	20751900	17176898	17096531	82.385377	324636	323945	1.8948	157516	48.6243	0.75904375
A35	shotgun	6138409	6077057	6037568	98.357213	52575	52385	0.867651	26906	51.36203	0.43832205
A36	capture	26757470	15193983	15101232	56.437443	658788	657269	4.35242	393807	59.91565	1.47176471
A36	shotgun	4672799	4635005	4602735	98.500599	81198	80989	1.759584	51054	63.03819	1.09257856
A37	capture	52676148	34547384	34378178	65.263272	486568	485230	1.411448	242139	49.9019	0.45967484
A37	shotgun	7168543	7117719	7076201	98.711844	50862	50676	0.716147	26808	52.90078	0.37396721
All		308167376	190800088								

Table S5. Number and frequencies of modern native Israeli sequences reads in the processing and mapping stages. Colours are relative to amounts in the same column with green being higher number/percentage of reads, orange and yellow are midrange and red is low.

	Preprocessing							Mapping				
**sample**	**Pairs of Raw Reads**	**After dup removal**	**After trimming paired**	**paired %**	**After trimming unpaired**	**percentage after trimming**	**unpaired %**	**mapped**	**mapped %**	**Mapped after filtering**	**% filtered mapped**	**% mapped from raw**
**GB80**	60584930	1994753	1514563	75.9273	3523473	84.75922	8.83185	2964242	87.6612	15342477	51.7585	12.66196
**GB81**	22564404	14687821	10828321	73.7231	3460564	85.50351	11.780	23473656	93.456	14255111	60.72812	31.58761
**GB84**	31345043	17112228	9904566	57.880	5218314	73.12737	15.247	16769873	67.005	5531702	32.98595	8.823886
**GB85**	18027632	7764480	5644853	72.700	1127247	79.95998	7.2589	8303765	66.874	2387062	28.74674	6.620564
**GB92**	82693248	30096824	20390336	67.749	7405231	80.05147	12.302	39552635	82.083	19655847	49.69542	11.8848
**GB94**	455216	321700	209997	65.277	80050	77.71899	12.441	367980	73.589	137497	37.36535	15.10239
**GB95**	352498	191511	100185	52.312	27709	59.54723	7.23431	161621	70.861	72198	44.67118	10.24091
**GB97**	132832	54010	35002	64.806	9177	73.30217	8.4956	61916	78.195	31543	50.94483	11.87327
**GB102**	56608	27192	14354	52.787	6253	64.28545	11.497	25667	73.416	11982	46.68251	10.58331
**GB103**	9110	8253	2086	25.275	4090	50.05453	24.778	5361	64.887	2491	46.46521	13.67179
**GB105**	932549	596937	123641	20.712	334622	48.74082	28.028	439770	75.574	152211	34.6115	8.161019
**GB106**	13539396	7030474	3365430	47.869	2724423	67.24499	19.375	6497286	68.715	2044463	31.46642	7.550052
**GB107**	52890302	16371461	10881483	66.466	4412288	79.94172	13.4755	21671581	82.794	11011519	50.81087	10.40977
**GB108**	65746400	22762551	16090103	70.686	4914540	81.48196	10.795	29714411	80.1041	14060229	47.31788	10.69277
**GB109**	20609333	8359519	5822763	69.654	1078105	76.10265	6.4483	8429052	66.247	2465459	29.24954	5.981414
**GB110**	272	198	54	27.272	2	27.77778	0.5050	86	78.181	47	54.65116	8.639706
**GB111**	7465	3961	2883	72.7846	310	76.6978	3.9131	4797	78.9499	2649	55.22201	17.7428
**GB112**	3371	2329	1443	61.957	709	77.17905	15.221	2723	75.744	1390	51.04664	20.61703
All	3.7E+08	1.45E+08	98563137	67.816	343271		11.809	1.85E+08	79.983	87165877	47.08498	11.78075

Table S6. Counts and frequencies of each type of substitution between the reference genome and the ancient samples (A31, A32, A33) in each processing stage

substitution		1st mapping		2nd mapping		filtered mapped		Genotyping		Filtered genotype		Genotype in 6,928 out of 10K SNPs	
**ref**	**alt**	**count**	**percent**	**count**	**percent**	**count**	**percent**	**count**	**percent**	**count**	**percent**	**count**	**percent**
A	C	62396	3.14	67784	4.54	65136	4.54	12046	4.742	2176	4.094	202	5.26
A	G	259574	13	282036	18.9	268163	18.7	49103	19.33	9865	18.56	784	20.4
A	T	84846	4.26	92431	6.2	89675	6.25	15341	6.038	2256	4.244	0	0
C	A	70437	3.54	77044	5.16	74412	5.19	12110	4.767	2232	4.199	210	5.47
C	G	42848	2.15	46759	3.13	45227	3.15	8346	3.285	1339	2.519	0	0
C	T	473071	23.8	178245	11.9	173695	12.1	29977	11.8	8748	16.46	706	18.4
G	A	474012	23.8	179190	12	174482	12.2	30365	11.95	8853	16.66	730	19
G	C	43098	2.17	46888	3.14	45371	3.16	8153	3.209	1320	2.483	0	0
G	T	70197	3.53	76515	5.13	73913	5.15	12122	4.771	2278	4.286	213	5.55
T	A	85126	4.28	92752	6.22	89955	6.27	15104	5.945	2264	4.259	0	0
T	C	261653	13.1	283728	19	269259	18.8	49279	19.4	9614	18.09	780	20.3
T	G	62962	3.16	68359	4.58	65631	4.57	12108	4.766	2207	4.152	214	5.57
sum		1990220		1491731		1434919		254054		53152		3839	

Table S7. Counts and frequencies of each type of substitution between the reference genome and nine capture sequenced modern samples in each processing stage

substitution		mapped		filtered mapped		Genotyping		Filtered genotype		Genotype in 6,928 out of 10K SNPs	
**ref**	**alt**	**count**	**percent**	**count**	**percent**	**count**	**percent**	**count**	**percent**	**count**	**percent**
A	C	107983	4.751	48388	4.267	21265	4.116	3650	4.607	277	5.039
A	G	354314	15.59	184324	16.25	80038	15.49	11492	14.5	1095	19.92
A	T	151142	6.65	63738	5.621	29040	5.621	4536	5.725	0	0
C	A	118852	5.229	53779	4.743	24899	4.819	4304	5.432	303	5.512
C	G	75565	3.325	33411	2.946	15845	3.067	2663	3.361	0	0
C	T	327144	14.39	183200	16.16	86581	16.76	12840	16.21	1064	19.36
G	A	328487	14.45	184078	16.23	87018	16.84	12992	16.4	1070	19.47
G	C	75943	3.341	33314	2.938	15624	3.024	2548	3.216	0	0
G	T	118137	5.198	52695	4.647	24783	4.797	4342	5.48	300	5.458
T	A	150269	6.612	64049	5.648	29245	5.661	4457	5.625	0	0
T	C	356447	15.68	185061	16.32	80501	15.58	11563	14.59	1092	19.87
T	G	108512	4.774	47917	4.226	21796	4.219	3842	4.849	296	5.385
sum		2272795		1133954		516635		79229		5497	

Figures

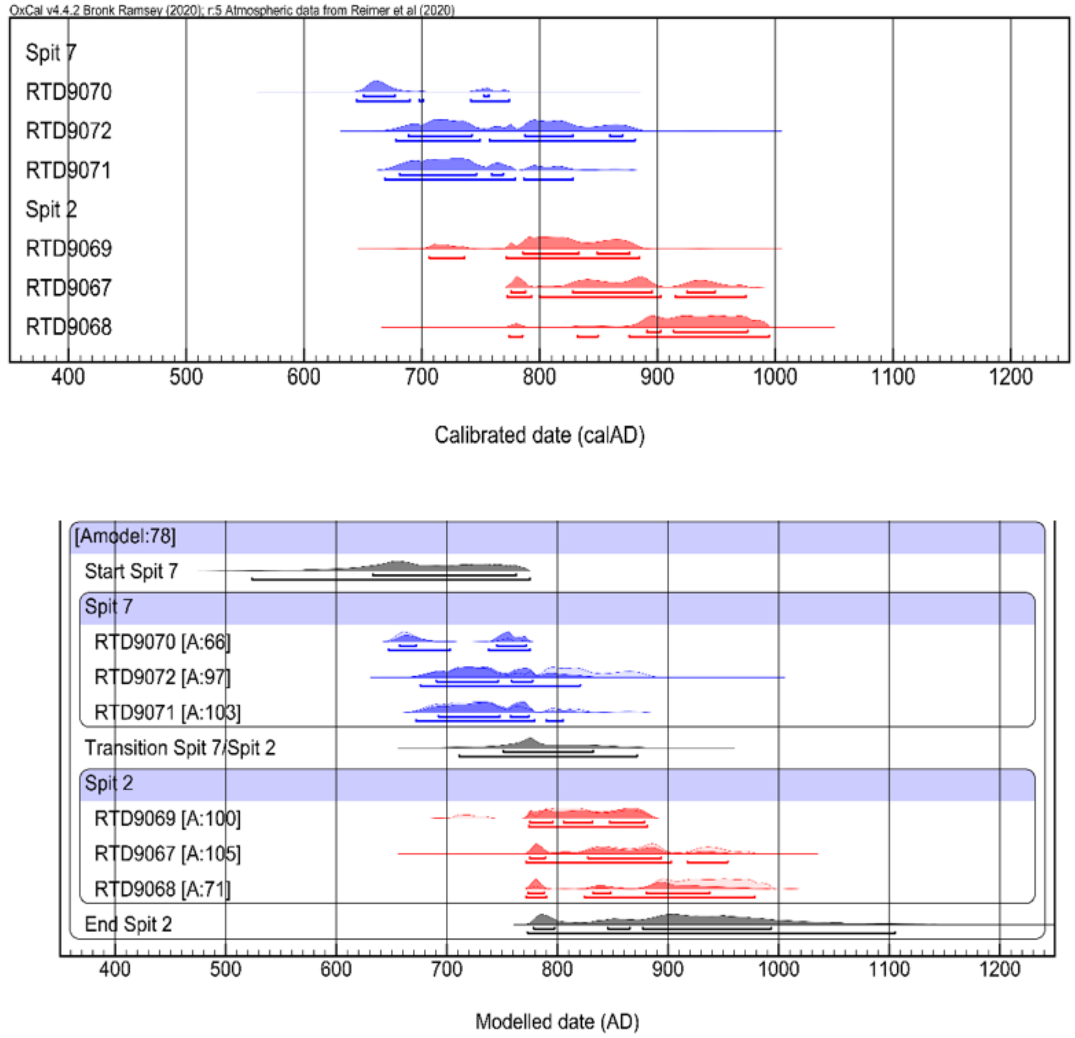

Figure S1. Radiocarbon probability distribution of the calibrated dates of the grape pips from Avdat. Top: The probability distribution of the calibrated dates of the grape pips. The dates are ordered by spit and in spit they are ordered from old to young. Bottom: Modelled probability distribution of the calibrated dates. The model is a sequence of two phases (spit 7 is older than spit 2).

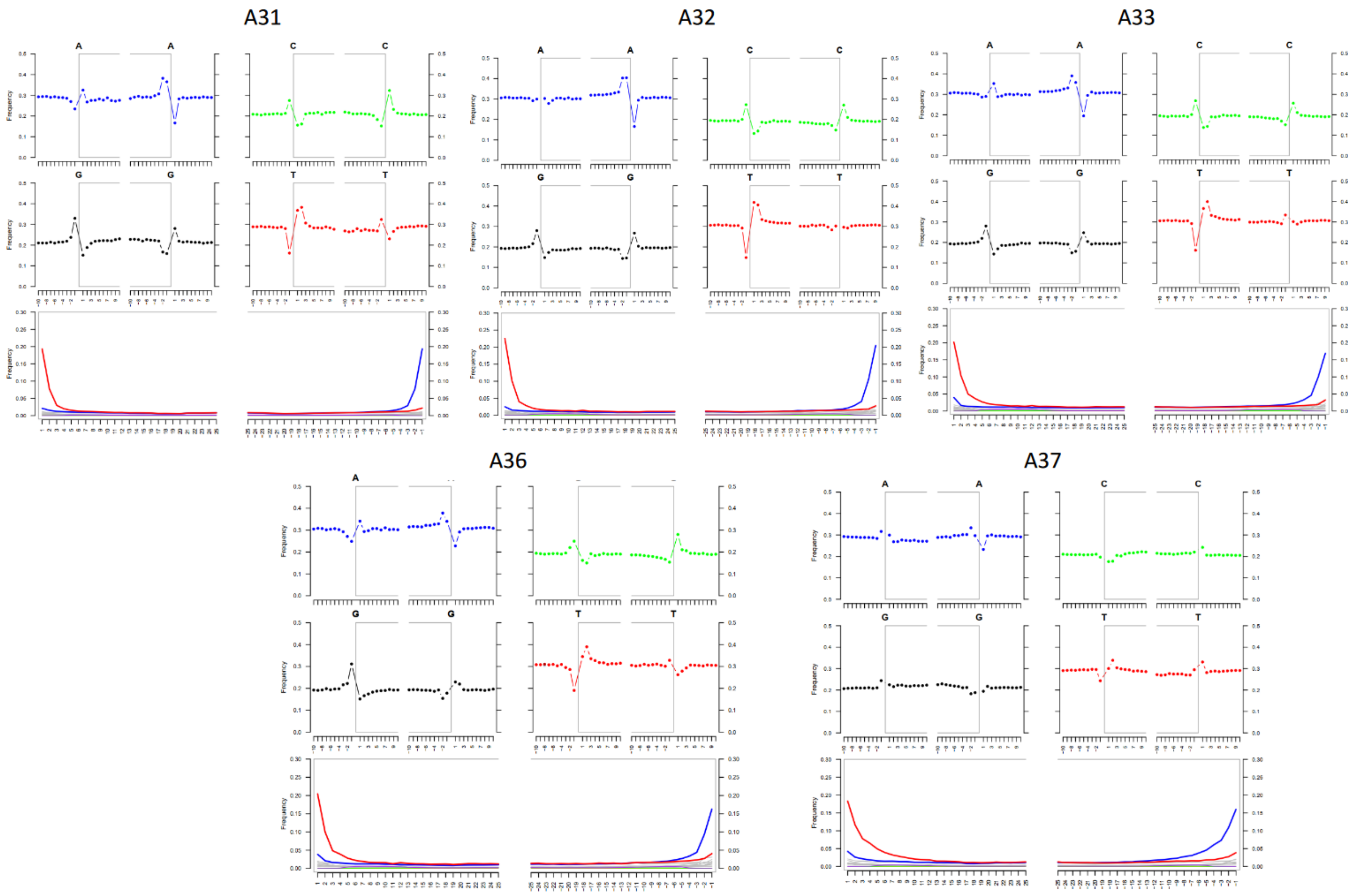

Figure S2. Patterns of deamination damage on sequences of the ancient, as detected by mapDamage software. X-axis: location on the sequence segment. Y-axis: substitution frequency.

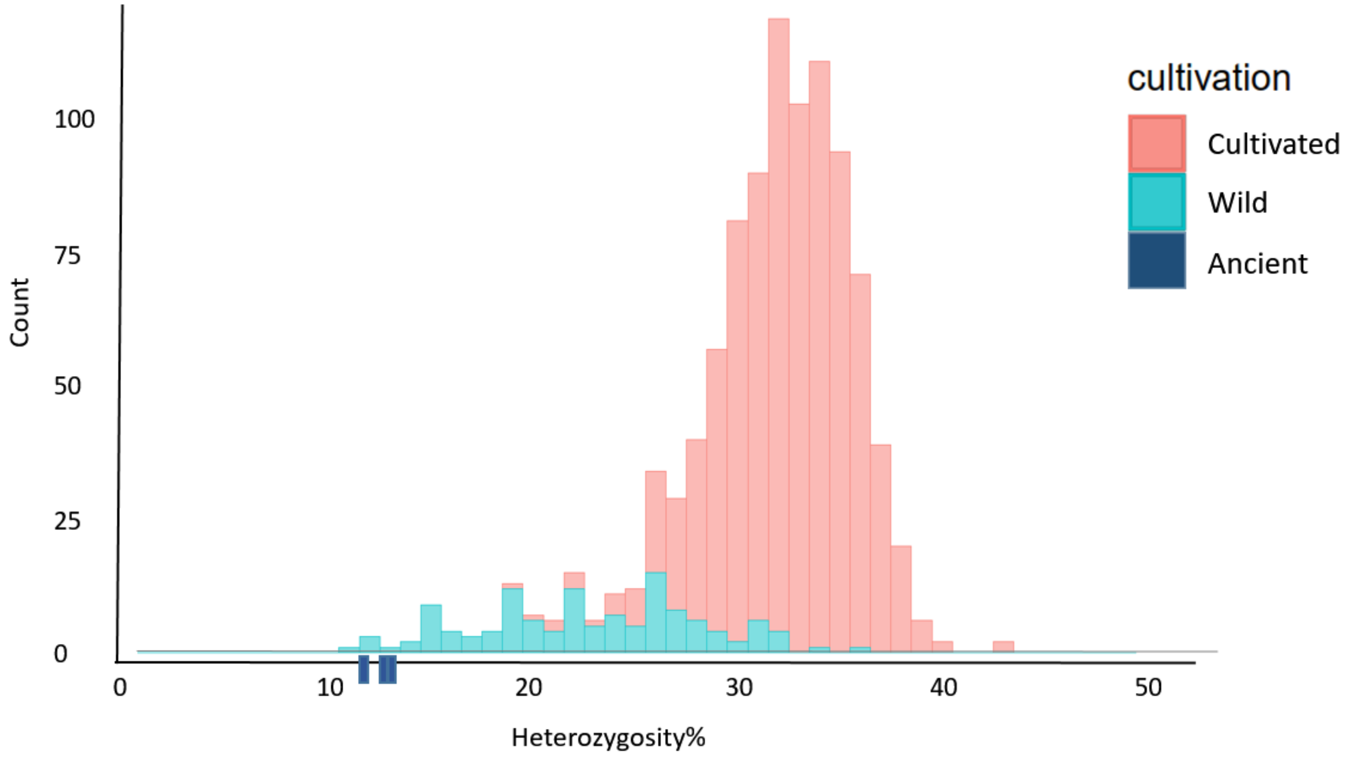

Figure S3. Heterozygosity percentage of cultivated (pink), wild (light blue) and three archaeological samples A31, A32 and A33 (dark blue) calculated over the large SNPs dataset of 6,928 SNPs. The archaeological samples are the least heterogenic as a group, however they fit within the distribution of heterozygosity in the wild group.

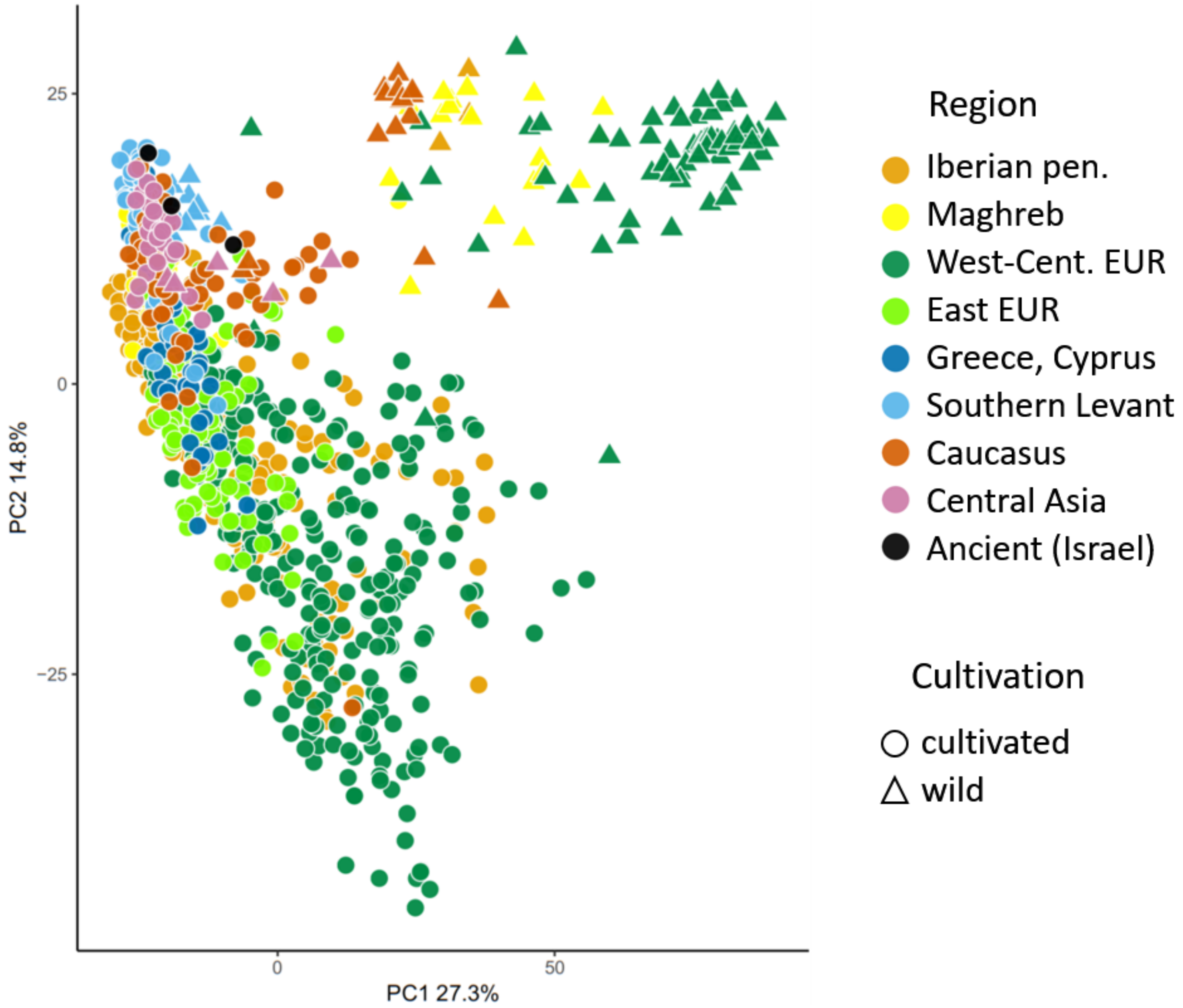

Figure S4. PCA plot of grapevine accessions across Eurasia executed over the large SNPs dataset. The geographic origin of the samples is colour coded as explained in the legend. Each sample is also classified by its cultivation status: cultivated in circle and wild in triangle.

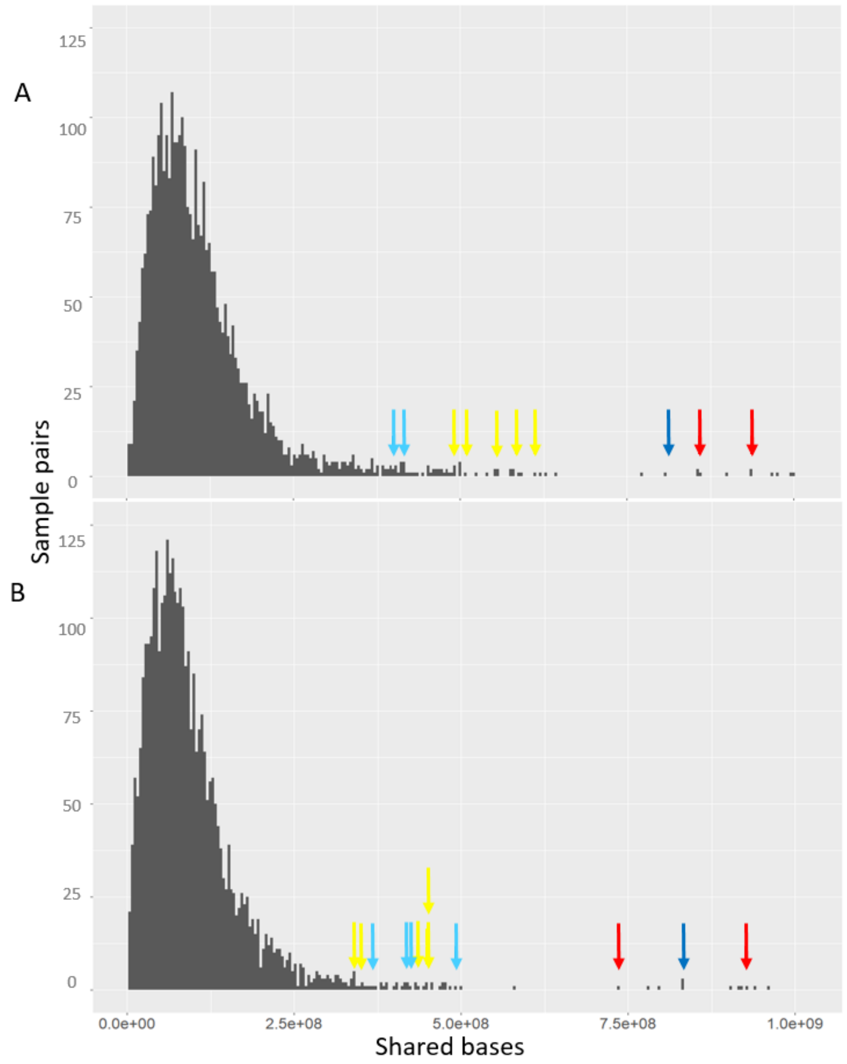

Figure S5. Summed length of shared IBD segments between all southern Levant sample pair combinations for the larger SNPs dataset (A) and the smaller SNPs dataset (B). Coloured arrows: In red are all known clones; in yellow all known parent-offspring pairs; in dark blue A33-*Aswad Karech* and in light blue all other purported kinships with an archaeological sample, from right to left: A31-A32, A33-A37 (only in B), A37-*Asswad Karech* (only in B), A31-*Be'er*

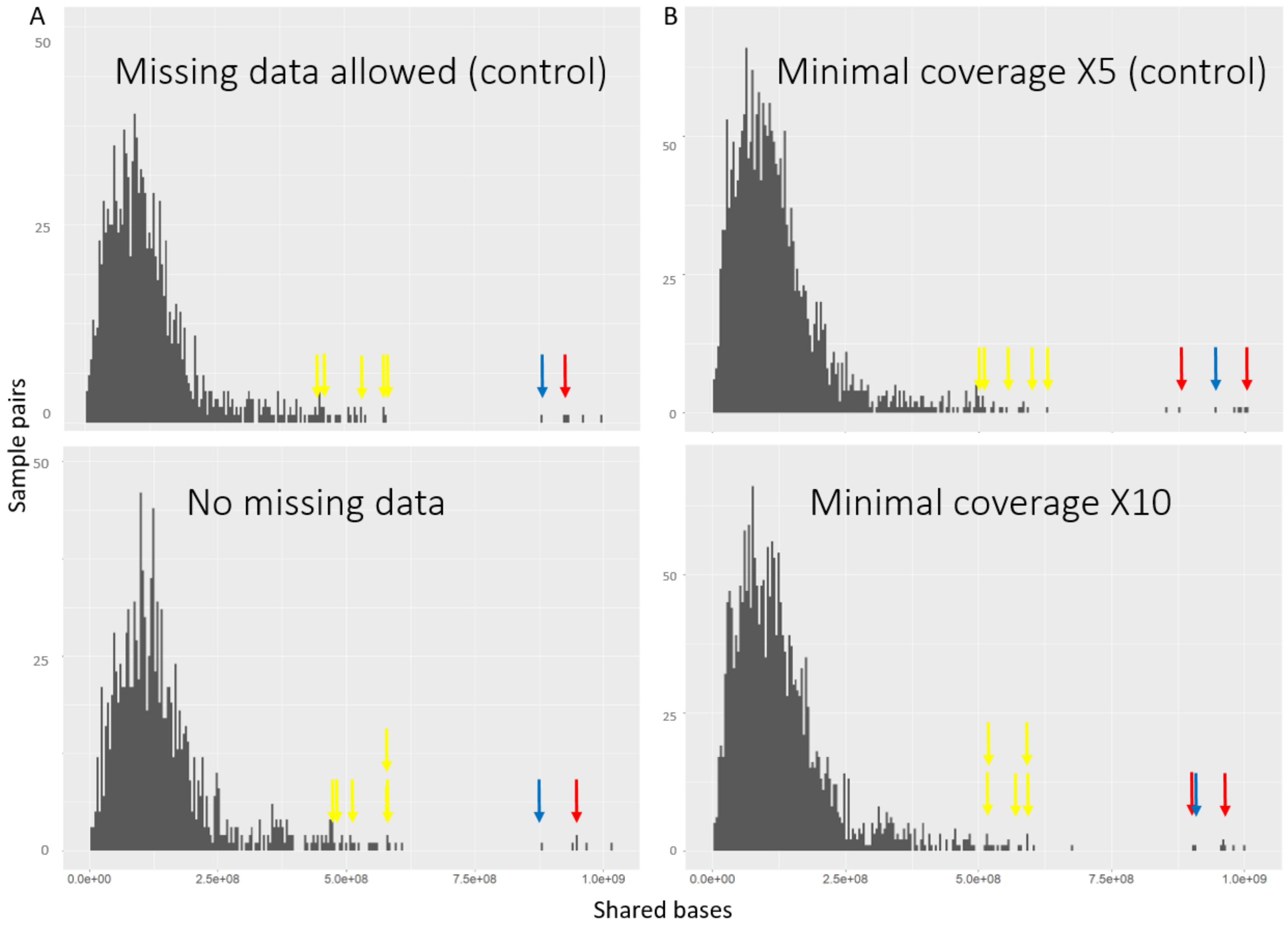

Figure S6. Summed length of shared IBD segments between in error control analyses. (A) bottom: no missing data allowed (50 samples and 5,146 SNPs) (B) bottom: minimal X10 coverage. Above are control analyses for the same samples and the same number of SNPs, respective of analyses, and no other limitations. All known clones and all known parent-offspring pairs included are marked with red and yellow arrows, respectively; Asswad Karech-A33 marked with a blue arrow.

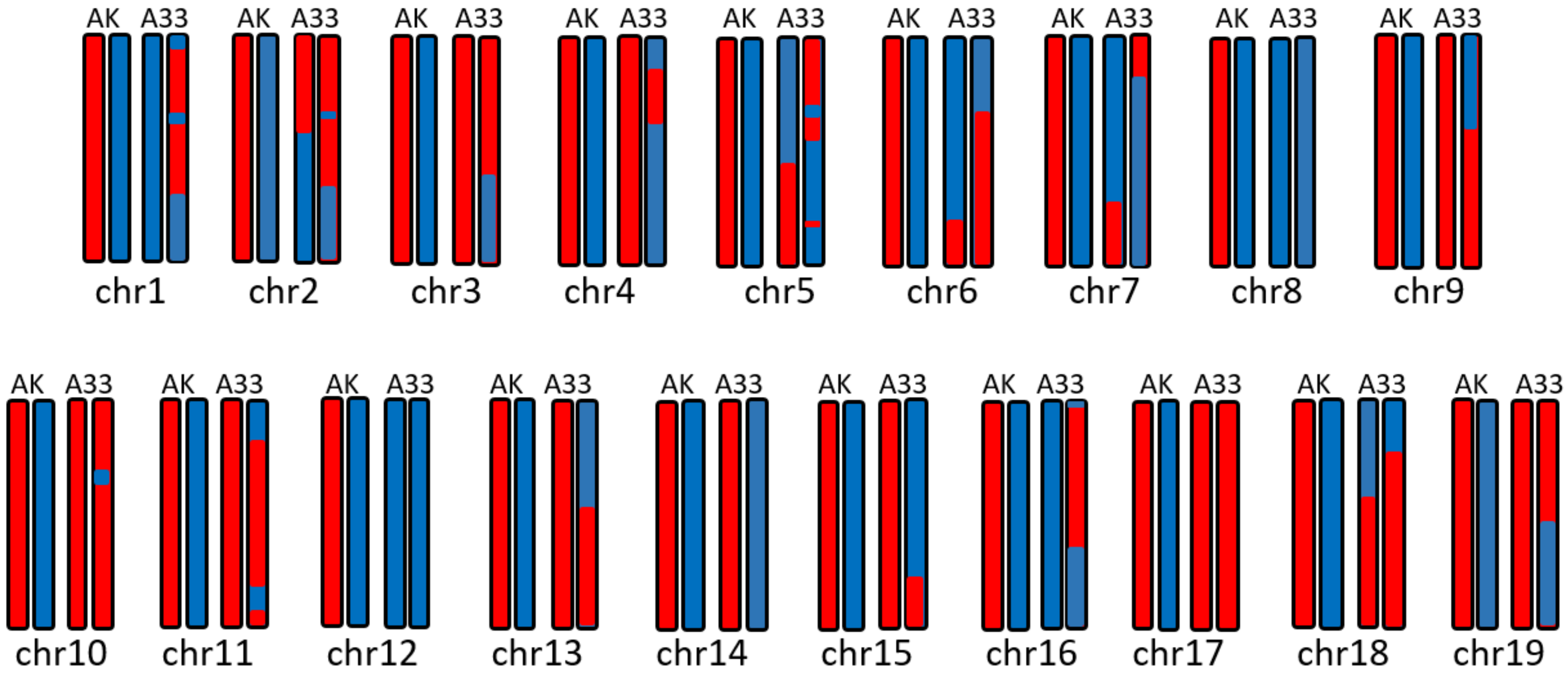

Figure S7. Reconstructed shared IBD segments between Asswad Karech (two leftmost) and A33 (two rightmost) across all 19 chromosomes in the grapevine genome. The colours represent the matching of the left chromosomal copy of *Asswad Karech* (in red) and of the right copy of *Asswad Karech* (in blue) to A33's two copies.

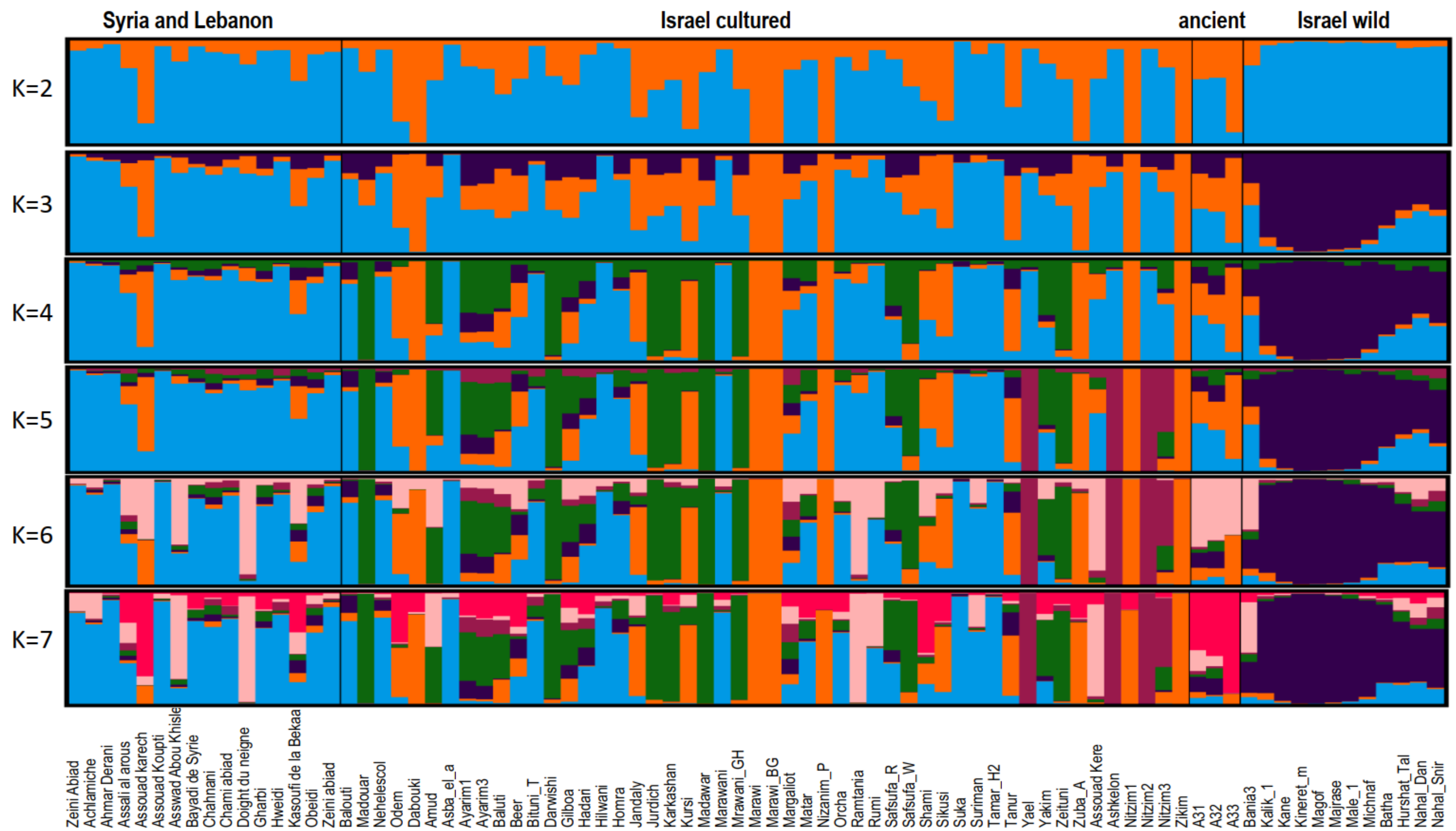

Figure S8. The consensus clustering of 100 *STRUCTURE* runs per K for K=2-7. Samples are divided to groups according to geographic origin and cultivation status (cultivated/wild). The native Israeli samples whose species is not known were classified here in accordance to the PCA results in Figure 3C. For each K, the archaeological samples are clustered in a similar fashion to the cultivated group

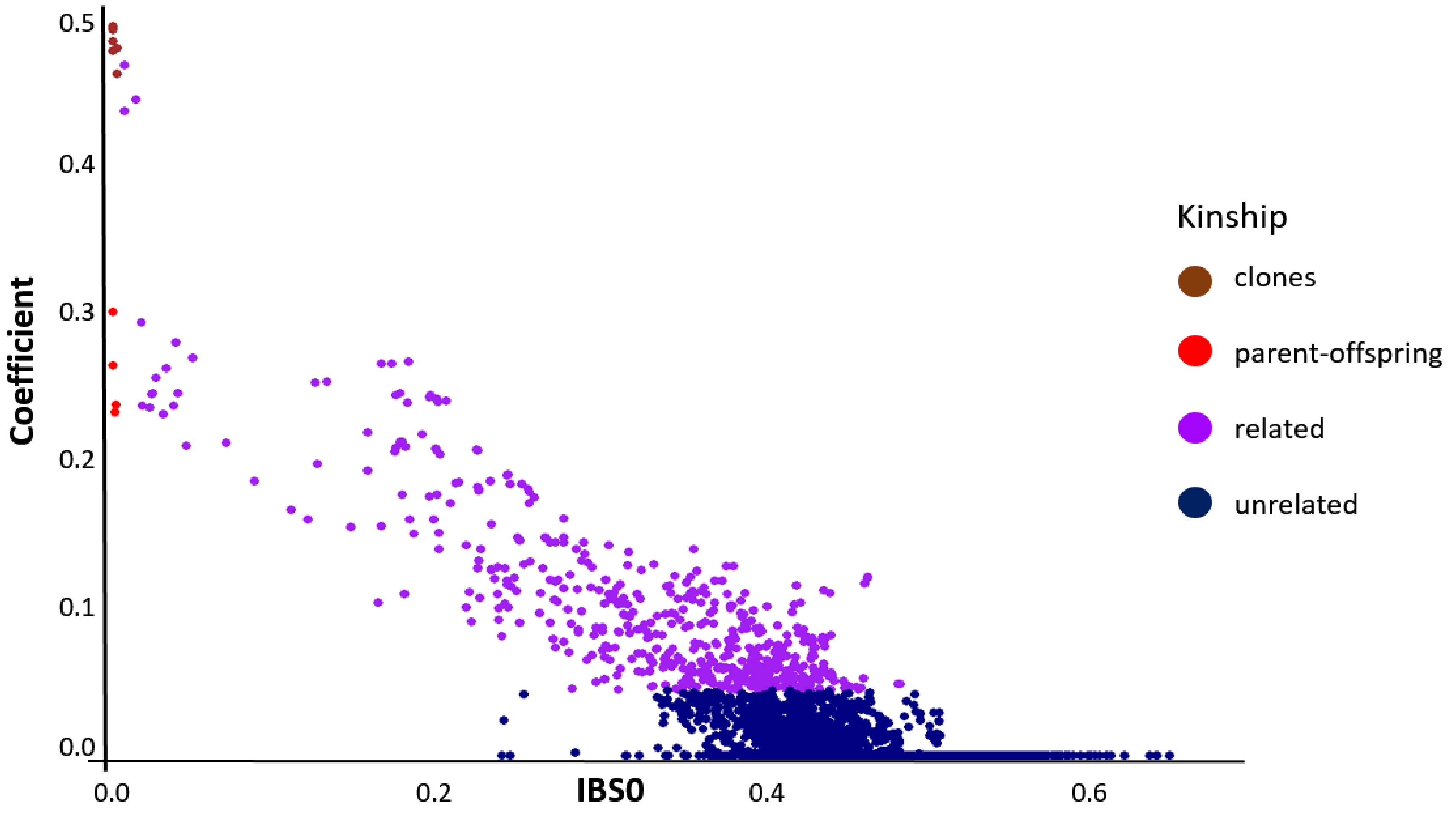

Figure S9. Relatedness between all Levant samples which was obtained using KING and plotted based on the conservative relatedness categories defined by Laucou *et al.* (2018)(30). K was set to 0 when the resulting kinship coefficient were negative.

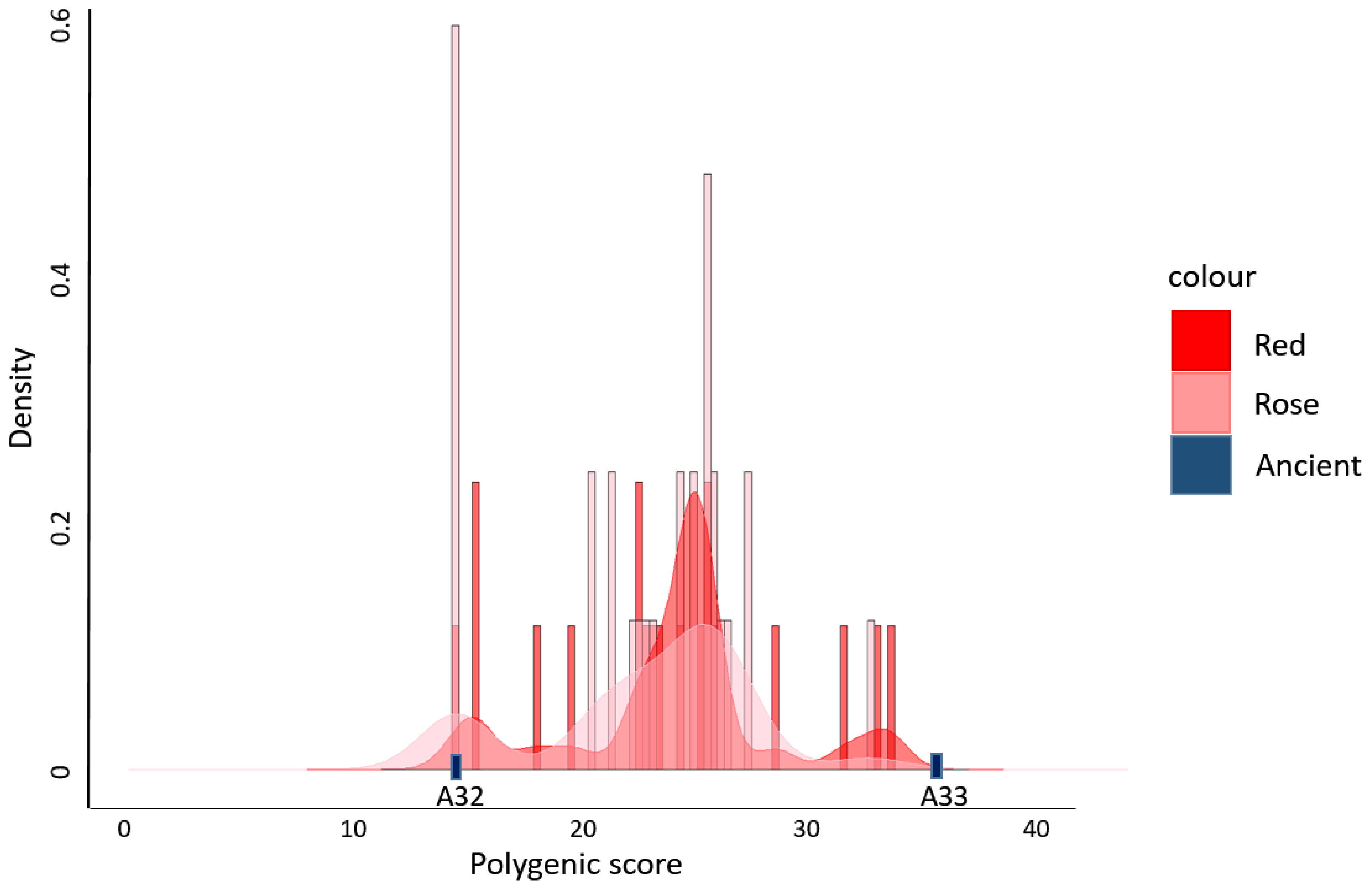

Figure S10. Histogram and density of the combined genomic effect (polyogenic score) of nineteen genomic loci associated with colour for 28 rosé, 29 red modern accessions, and the combined genomic effect of two archaeological samples, A32 and A33.

References

1. G. Bar-Oz, *et al.*, Ancient trash mounds unravel urban collapse a century before the end of Byzantine hegemony in the southern Levant. *PNAS* **116**, 8239–8248 (2019).

2. D. H. Butler, *et al.*, Byzantine - Early Islamic resource management detected through micro-georchaeological investigations of trash mounds (Negev, Israel). *PLoS One* **15** (2020).

3. E. Fuks, D., Bar-Oz, G., Tepper, Y., Erickson-Gini, T., Langgut, D., Weissbrod, L., & Weiss, *et al.*, The rise and fall of viticulture in the Late Antique Negev Highlands reconstructed from archaeobotanical and ceramic data. *Proc. Natl. Acad. Sci. U. S. A.* **117**, 19780–19791 (2020).

4. N. Marom, *et al.*, Zooarchaeology of the social and economic upheavals in the Late Antique-Early Islamic sequence of the Negev Desert.No Title. *Sci. Rep.* **9**, 1–10 (2019).

5. E. W. D. Fuks, G. Bar-Ozb, Y. Tepper, T.Erickson-Ginic, D. Langgutd, L. Weissbrod, The rise and fall of viticulture in the Late Antique Negev Highlands reconstructed from archaeobotanical and ceramic data. *PNAS* (2019).

6. S. Bucking, D. Fuks, Z. C. Dunseth, L. Schwimer, T. Erickson-Gini, The Avdat in Late Antiquity Project : uncovering the Early Islamic phases of a Byzantine town in the Negev Highlands . *Antiquity*, 1–8 (2022).

7. Y. Tepper, L. Weissbrod, G. Bar-Oz, Behind sealed doors: Unraveling abandonment dynamics at the Byzantine site of Shivta in the Negev Desert. *Antiq. (Project Gall.* **348**, 1–4 (2015).

8. Y. Tepper, T. Erickson-Gini, Y. Farhi, G. Bar-Oz, Probing the Byzantine/Early Islamic Transition in the Negev: The renewed Shivta excavations, 2015-2016. *Tel Aviv* **45**, 120–152 (2018).

9. Y. Tepper, L. Weissbrod, T. Erickson-Gini, G. Bar-Oz, Nessana: A preliminary report. Excavations and surveys in Israel. *Excav. Surv. Isr.* (2020).

10. S. Bucking, T. Erickson-Gini, The Avdat in late antiquity project: Report on the 2012/2016 excavations of a cave and stone-built compound along the southern slope. *J. East. Mediterr. Archeol. Herit. Stud.* **8** (2020).

11. M. Creisher, Y. Goren, M. Artzy, D. Cvikel, The amphorae of the Ma'agan Mikhael B shipwreck: preliminary report. *Levant* **51**, 105–120 (2019).

12. M. Cohen, D. Cvikel, Rigging of the Ma 'agan Mikhael B shipwreck (7th–8th centuries AD): new finds. *Int. J. Naut. Archaeol.* **49**, 291–302 (2020).

13. G. Besnard, *et al.*, History of the invasive African olive tree in Australia and Hawaii: Evidence for sequential bottlenecks and hybridization with the Mediterranean olive. *Evol. Appl.* **7**, 195–211 (2014).

14. J. Dabney, *et al.*, Complete mitochondrial genome sequence of a Middle Pleistocene cave bear reconstructed from ultrashort DNA fragments. *Proc. Natl. Acad. Sci. U. S. A.* **110**, 15758–15763 (2013).

15. and M. T. P. G. Wales, Nathan, Christian Carøe, Marcela Sandoval-Velasco, Cristina Gamba, Ross Barnett, José Alfredo Samaniego, Jazmín Ramos Madrigal, Ludovic Orlando, ssDNA vs dsDNA library prep. *Biotechniques* **59** (2015).

16. C. Carøe, *et al.*, Single-tube library preparation for degraded DNA. *Methods Ecol. Evol.* **9**, 410–419 (2018).

17. M. Meyer, M. Kircher, Illumina sequencing library preparation for highly multiplexed target capture and sequencing. *Cold Spring Harb. Protoc.* **5** (2010).

18. J. Ramos-Madrigal, *et al.*, Palaeogenomic insights into the origins of French grapevine diversity. *Nat. Plants* **5**, 595–603 (2019).

19. K. M. Meyer M, llumina sequencing library preparation for highly multiplexed target capture and sequencing. *Cold Spring Harb Protoc. Protoc.* **6** (2010).

20. G. Renaud, U. Stenzel, J. Kelso, LeeHom: Adaptor trimming and merging for Illumina sequencing reads. *Nucleic Acids Res.* **42**, e141 (2014).

21. A. M. Bolger, M. Lohse, B. Usadel, Trimmomatic: A flexible trimmer for Illumina sequence data. *Bioinformatics* **30**, 2114–2120 (2014).

22. M. Poullet, L. Orlando, Assessing DNA Sequence Alignment Methods for Characterizing Ancient Genomes and Methylomes. *Front. Ecol. Evol.* **8** (2020).

23. K. G. et al. McKenna, Aaron, Matthew Hanna, Eric Banks, Andrey Sivachenko, Kristian Cibulskis, Andrew Kernytsky, The Genome Analysis Toolkit: A MapReduce framework for analyzing next-generation DNA sequencing data. *Genome Res.* **20**, 1297– 1303 (2010).

24. P. Danecek, *et al.*, The variant call format and VCFtools. *Bioinformatics* **27**, 2156–2158 (2011).

25. R. Martiniano, *et al.*, The population genomics of archaeological transition in west Iberia: Investigation of ancient substructure using imputation and haplotype-based methods. *PLoS Genet.* **13**, 1–24 (2017).

26. R. Martiniano, *et al.*, Genomic signals of migration and continuity in Britain before the Anglo-Saxons. *Nat. Commun.* **7**, 1–8 (2016).

27. C. Gamba, *et al.*, Genome flux and stasis in a five millennium transect of European prehistory. *Nat. Commun.* **5**, 1–9 (2014).

28. K. Ausmees, F. Sanchez-Quinto, M. Jakobsson, C. Nettelblad, An empirical evaluation of genotype imputation of ancient DNA. *G3 Genes, Genomes, Genet.* **12** (2022).

29. B. Sousa da Mota, *et al.*, Imputation of ancient genomes. *bioRxiv*, 2022.07.19.500636 (2022).

30. V. Laucou, *et al.*, Extended diversity analysis of cultivated grapevine Vitis vinifera with 10K genome-wide SNPs. *PLoS One*, 1–27 (2018).

31. T. Lacombe, *et al.*, Large-scale parentage analysis in an extended set of grapevine cultivars (Vitis vinifera L.). *Theor. Appl. Genet.* **126**, 401–414 (2013).

32. R. Bacilieri, *et al.*, Genetic structure in cultivated grapevines is linked to geography and human selection. *BMC Plant Biol.* **13** (2013).

33. K. N. M., *et al.*, Clumpak: A program for identifying clustering modes and packaging population structure inferences across K. *Mol. Ecol. Resour.* **8**, 1179–1191 (2015).

34. B. L. Browning, S. R. Browning, Improving the accuracy and efficiency of identity-by-descent detection in population data. *Genetics* **194**, 459–471 (2013).

35. A.-B. A. Le Paslier MC, Choisne N, Bacilieri R, Boursiquot, Bras M, Brunel D, Chauveau A, Hausmann L, Lacombe T, Laucou V, Launay A, Marchal C, Martinez-Zapater JM, Berard A, Quesneville H, Tépfer R, Torres-Perez R, "A dataset of 9.896 single nuclear polymorphisms for 112 wild grapes, obtained with the GrapeReSeq 18K Vitis chip" (2019) https:/doi.org/https://doi.org/10.15454/9RUCEP.

36. A. Sivan, *et al.*, Genomic evidence supports an independent history of Levantine and Eurasian grapevines. *Plants People Planet* **3**, 414–427 (2021).

37. Y. Zhou, M. Massonnet, J. S. Sanjak, D. Cantu, B. S. Gaut, Evolutionary genomics of grape (Vitis vinifera ssp. vinifera) domestication. *Proc. Natl. Acad. Sci. U. S. A.* **114**, 11715–11720 (2017).

38. Z. Liang, *et al.*, Whole-genome resequencing of 472 Vitis accessions for grapevine diversity and demographic history analyses. *Nat. Commun.* **10**, 1–12 (2019).
